# Inferring microbial co-occurrence networks from amplicon data: a systematic evaluation

**DOI:** 10.1128/msystems.00961-22

**Published:** 2023-06-20

**Authors:** Dileep Kishore, Gabriel Birzu, Zhenjun Hu, Charles DeLisi, Kirill S. Korolev, Daniel Segrè

**Affiliations:** 1 Bioinformatics Program, Boston University, Boston, Massachusetts, USA; 2 Biological Design Center, Boston University, Boston, Massachusetts, USA; 3 Biosciences Division, Oak Ridge National Laboratory, Oak Ridge, Tennessee, USA; 4 Department of Physics, Boston University, Boston, Massachusetts, USA; 5 Department of Applied Physics, Stanford University, Stanford, California, USA; 6 Department of Biomedical Engineering, Boston University, Boston, Massachusetts, USA; 7 Department of Biology, Boston University, Boston, Massachusetts, USA; Katholieke Universiteit Leuven, Leuven, Belgium

**Keywords:** Microbiome, 16S rRNA, interaction, denoising, taxonomy, network inference, correlations, QIIME2, co-occurrence, networks, consensus algorithm, pipeline, nextflow

## Abstract

Microbes commonly organize into communities consisting of hundreds of species involved in complex interactions with each other. 16S ribosomal RNA (16S rRNA) amplicon profiling provides snapshots that reveal the phylogenies and abundance profiles of these microbial communities. These snapshots, when collected from multiple samples, can reveal the co-occurrence of microbes, providing a glimpse into the network of associations in these communities. However, the inference of networks from 16S data involves numerous steps, each requiring specific tools and parameter choices. Moreover, the extent to which these steps affect the final network is still unclear. In this study, we perform a meticulous analysis of each step of a pipeline that can convert 16S sequencing data into a network of microbial associations. Through this process, we map how different choices of algorithms and parameters affect the co-occurrence network and identify the steps that contribute substantially to the variance. We further determine the tools and parameters that generate robust co-occurrence networks and develop consensus network algorithms based on benchmarks with mock and synthetic data sets. The Microbial Co-occurrence Network Explorer, or MiCoNE (available at https://github.com/segrelab/MiCoNE) follows these default tools and parameters and can help explore the outcome of these combinations of choices on the inferred networks. We envisage that this pipeline could be used for integrating multiple data sets and generating comparative analyses and consensus networks that can guide our understanding of microbial community assembly in different biomes.

**IMPORTANCE:**

Mapping the interrelationships between different species in a microbial community is important for understanding and controlling their structure and function. The surge in the high-throughput sequencing of microbial communities has led to the creation of thousands of data sets containing information about microbial abundances. These abundances can be transformed into co-occurrence networks, providing a glimpse into the associations within microbiomes. However, processing these data sets to obtain co-occurrence information relies on several complex steps, each of which involves numerous choices of tools and corresponding parameters. These multiple options pose questions about the robustness and uniqueness of the inferred networks. In this study, we address this workflow and provide a systematic analysis of how these choices of tools affect the final network and guidelines on appropriate tool selection for a particular data set. We also develop a consensus network algorithm that helps generate more robust co-occurrence networks based on benchmark synthetic data sets.

## INTRODUCTION

Microbial communities are ubiquitous and play an important role in marine and terrestrial environments, urban ecosystems, and human health (1 - 7). These microbial communities, or microbiomes, often comprise several hundreds of different microbial strains interacting with each other and their environment, often through complex metabolic and signaling relationships (8 - 11). Understanding how these interconnections shape community structure and function is a fundamental challenge in microbial ecology and has applications in the study of microbial ecosystems across different biomes. With the advancement in DNA sequencing technologies (12 - 14), more information can be extracted from these microbial community samples than ever before. In particular, high-throughput sequencing, including metagenomic sequencing and sequencing of 16S ribosomal RNA (16S rRNA) gene amplicons (hereafter referred to as 16S data) of microbial communities, can help detect, identify, and quantify a large portion of the constitutive microorganisms of a microbiome (15 - 18). These advances have led to large-scale data collection efforts involving terrestrial (2, 4, 19), marine (1, 3), and human-associated microbiota (7, 20, 21).

This wealth of information has the potential to help us understand how communities assemble and operate. In particular, a powerful tool for translating microbiome composition data into knowledge is the construction of association (co-occurrence) networks, in which microbial taxa are represented by nodes, and frequent co-occurrences (or negative co-occurrences) across data sets are encoded as edges between nodes. While the relationship between directly measured interactions (22 - 24) and statistically inferred co-occurrence is still poorly understood (25, 26), a significant amount of effort has gone into estimating co-occurrences from large microbiome sequence data sets (27 - 30).

The importance of these networks is twofold: first, they can serve as maps that help identify hubs of keystone species (26, 31) and the community response to environmental perturbations or underlying host conditions (32); second, they can serve as a bridge toward building mechanistic models of microbial communities, greatly enhancing our capacity to understand and control them. For example, multiple studies have shown the importance of specific microbial associations in the healthy microbiome (7, 21, 33) and their role in dysbiosis (32, 34, 35). In the context of terrestrial biogeochemistry, co-occurrence networks were shown to help understand microbiome assembly (36) and the response of microbial communities to environmental perturbations (37).

One of the most frequently used avenues for inferring co-occurrence networks is the parsing and analysis of 16S sequencing data (26, 38). Numerous software tools and pipelines have been developed to analyze 16S sequencing data, with a strong emphasis on the known limitations of this method, including resolution, sequencing depth, compositional nature, sequencing errors, and copy number variations (39, 40). Popular methods for different phases of the analysis of 16S data include tools for (i) quality checking and trimming the sequencing reads, (ii) denoising and clustering the trimmed reads (41 - 43), (iii) assigning taxonomy to the denoised reads (44), (iv) processing and transforming the taxonomy count matrices (45), and (v) inferring the co-occurrence network (46 - 48). Different specific algorithms are often aggregated into popular online platforms (such as MG-RAST (49) and Qiita (50)) and software packages (such as Quantitative Insights Into Microbial Ecology 2 (QIIME2) (51)). The different methods and tools can lead to vastly different inferences of community compositions and co-occurrence networks (52, 53), making it difficult to reliably compare networks across different publications and studies. This difference is partially due to the focus of existing platforms on operational taxonomic unit (OTU) or exact sequence variant (ESV) generation and not on the effects of upstream statistical methods on the inferred co-occurrence networks. Furthermore, no organized framework currently exists that can systematically analyze and compare each step in the pipeline for processing amplicons into co-occurrence networks.

In this study, we present a standardized 16S data analysis pipeline called Microbial Co-occurrence Network Explorer (MiCoNE) that produces robust and reproducible co-occurrence networks from 16S sequence data of microbial communities and enables users to interactively explore how the network would change upon using different alternative tools and parameters at each step. Our pipeline is coupled to an online integrative tool for the organization, visualization, and analysis of inter-microbial networks called Microbial Interaction Network Database (MIND) (54), which is available at http://microbialnet.org/. Through a systematic comparative analysis, we determine which steps of the MiCoNE pipeline have the largest influence on the final network and which choice seems to have the optimal agreement with the tested mock and synthetic data sets. These steps together with our default settings ensure better reproducibility and easier comparison of co-occurrence networks across data sets. We expect that our tool will also be useful for benchmarking future alternative methods and ensuring a transparent evaluation of the possible biases introduced by the use of specific tools.

## RESULTS

### MiCoNE

**Fig 1:**
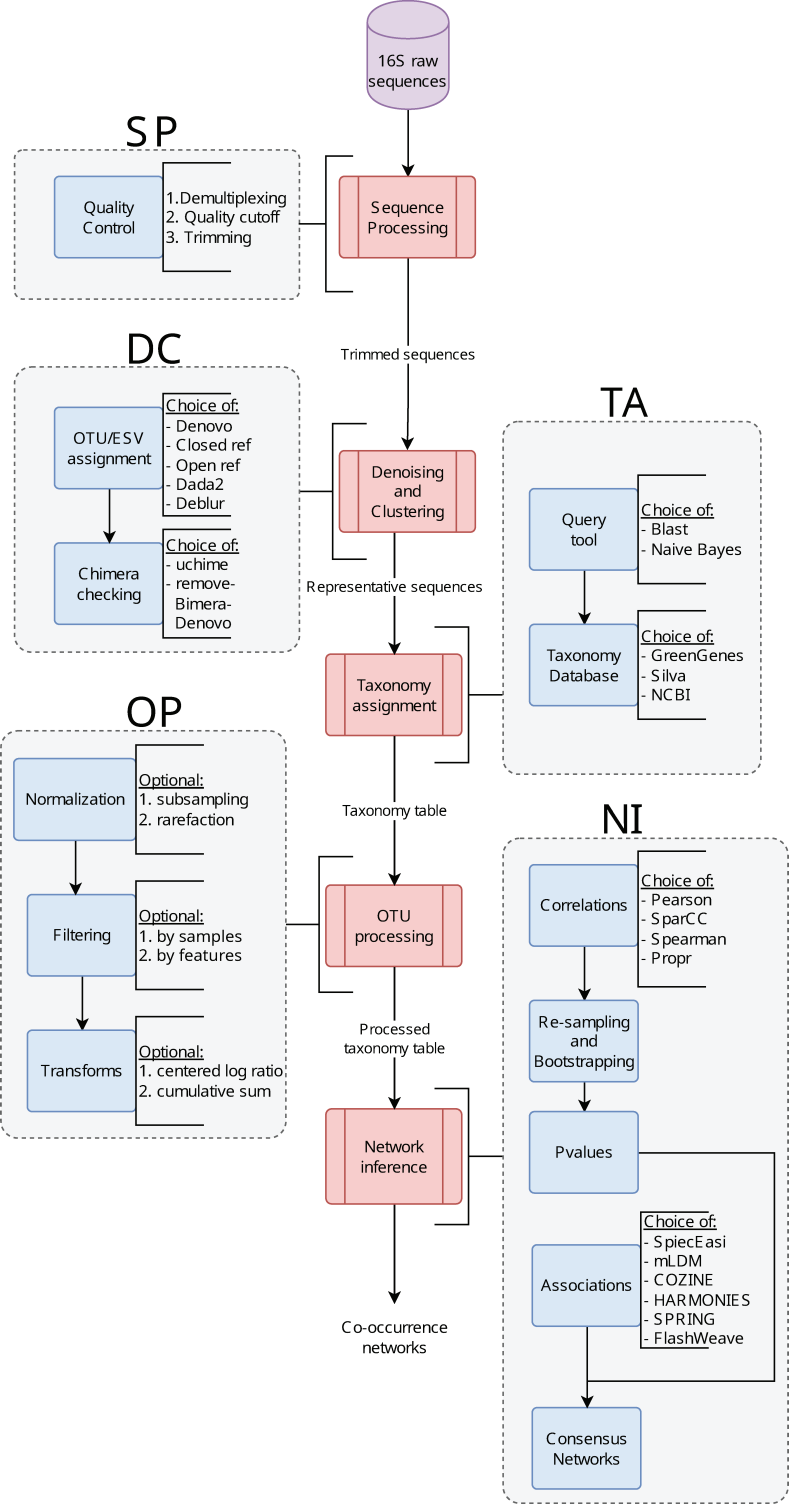
The workflow of the MiCoNE pipeline. The steps of the workflow can be broken down into five major groups: (SP) sequence processing, (DC) denoising and clustering, (TA) taxonomy assignment, (OP) OTU and ESV processing, and (NI) network inference. Each step incorporates several processes (blue boxes), each of which, in turn, has several alternative algorithms for the same task (indicated by the text to the right of the blue boxes). Each arrow describes the data that is being passed from one step to another. The inputs to the pipeline are 16S rRNA sequencing reads, and the final output is the consensus network generated from the inferred co-occurrence networks. For details on each process and the different outputs, see Methods.

We developed MiCoNE, a flexible and modular pipeline for the inference of co-occurrence networks from 16S data. MiCoNE incorporates various popular, publicly available tools as well as custom Python modules for 16S data analysis and network inference (see Methods). The different steps that are a part of the MiCoNE co-occurrence network inference workflow (Fig. 1) can be grouped into five major modules: (i) sequence processing (SP), (ii) Denoising and Clustering (DC), (iii) taxonomy assignment (TA), (iv) OTU processing (OP), and (v) network inference (NI). Each module in the pipeline is implemented through multiple tools (see Methods and Fig. 1). The effects of changing any intermediate step of the pipeline can be evaluated in terms of the final network outcome as well as on any of the intermediate metrics and data outputs. The choice of tools and parameters is encoded in a configuration file (with parameters as shown in Tables S2–S6 at https://github.com/segrelab/MiCoNE-pipeline-paper). Through a systematic analysis of tool combinations at each step of the pipeline, we estimated how much the final co-occurrence network depends on the possible choices at each step.

Our analysis involved two types of data: The first type consisted of 16S sequencing data from samples of human stool microbiomes from a fecal microbiome transplant (FMT) study of autism (55). The second type was a collection of data sets synthetically or artificially created for the specific goal of evaluating computational analysis tools. In particular, in order to benchmark each step in MiCoNE, we used both mock data (labeled mock4, mock12, and mock16) from mockrobiota (56) and synthetic networks generated using the NorTA (47) and seqtime (26) approaches (see Methods).

### DC: Denoising and clustering methods differ in their identification of sequences that are low in abundance

The DC step is commonly carried out to generate representative sequences (in the form of OTU/ESV tables) from the demultiplexed and trimmed 16S sequencing data. In order to compare the count tables generated by different tools, we processed the 16S sequencing reads (from the FMT study (55)) using five different methods: open-reference clustering, closed-reference clustering, *de novo* clustering, Divisive Amplicon Denoising Algorithm 2 (DADA2) (42), and Deblur (43). The first three methods are from the vsearch plugin from QIIME2 (51). The closed and open reference methods in this analysis use the Greengenes (57) database for reference sequence alignment.

**Fig 2:**
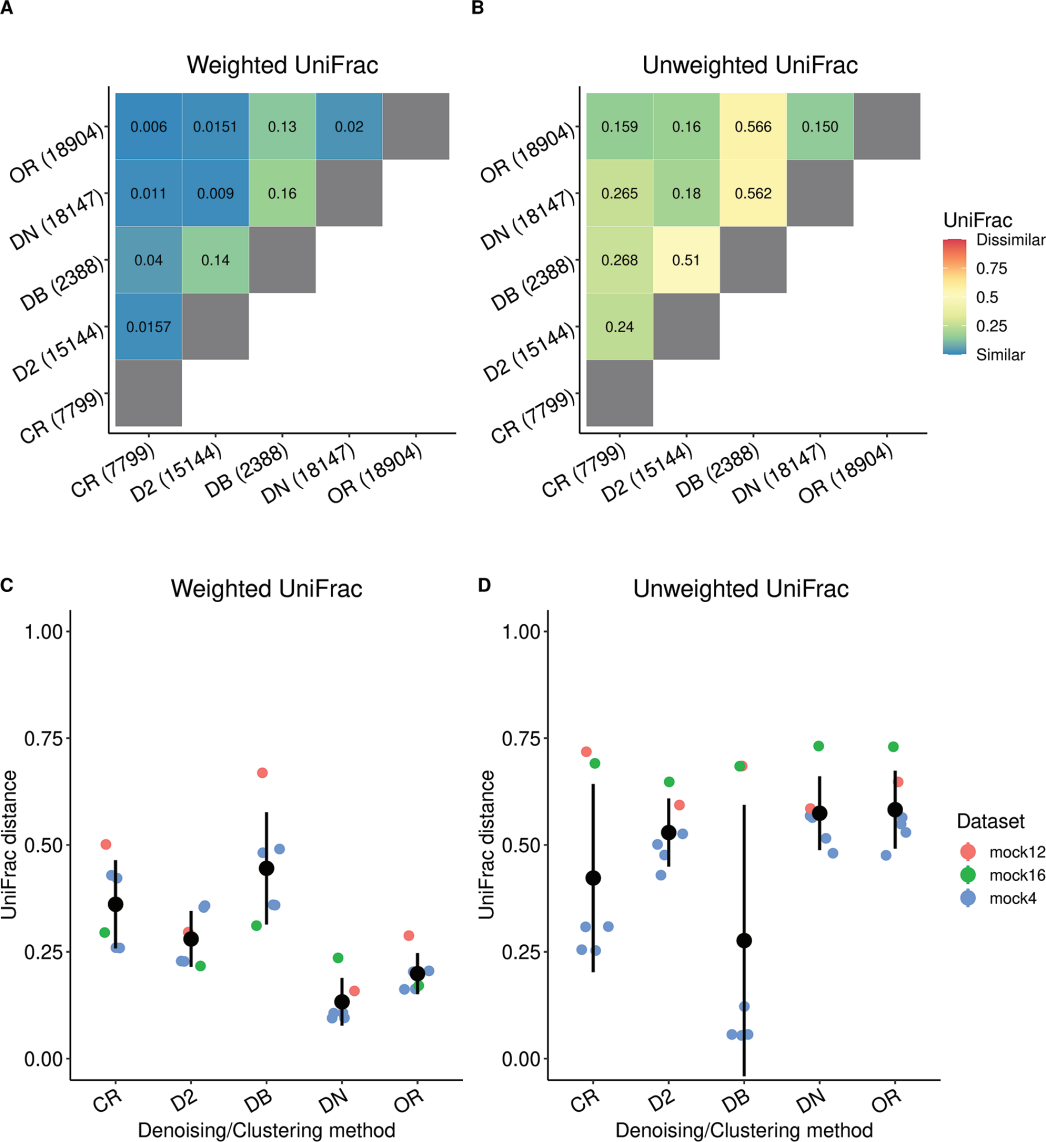
The representative sequences generated by the different denoising and clustering methods differ in their identification of sequences that are low in abundance. (**A**) The average weighted UniFrac distance between the representative sequences shows that the representative sequences and their compositions are fairly identical between the methods (with the exception of Deblur (DB) due to the low ESV count). (**B**) The relatively larger average unweighted UniFrac distance indicates that methods differ in their identification of sequences that are lower in abundance. The number of OTUs or ESVs generated by the respective methods is provided in the parenthesis next to their names. The data used for the analysis in (**A and B**) were the samples from the fecal microbiome transplant (FMT) data set (55), containing both healthy subjects and subjects with autism spectrum disorder (ASD). (**C and D**) The distributions of the average weighted and unweighted UniFrac distance between the predicted sequence profile and the expected sequence profile in the mock data sets. The average weighted UniFrac distances show that *de novo* (DN) and open reference (OR) were the best-performing methods in most of the data sets, while they are the worst-performing methods under the unweighted UniFrac metric. The good performance of DADA2 (D2) under both distance metrics combined with its approach of identifying ESVs using *de novo* methods prompts us to use it as the default method for the DC step. The data used for the analysis in (**C and D**) were the mock4, mock12, and mock16 data sets from mockrobiota (56).

A comparison of the different methods was carried out by calculating the mean UniFrac distances across all samples (Fig. 2). The analysis was performed using both the weighted UniFrac (58) (Fig. 2A) distance metric, which takes into account the counts of the representative sequences, and the unweighted UniFrac (59) (Fig. 2B) distance metric, which gives equal weights to each sequence.

The first main message emerging from this analysis is that the representative sequences generated by the different methods, with the exception of Deblur, are similar to each other when weighted by their abundance (Fig. 2A). A second message is that the different methods differ mainly in the assignment of sequences of lower abundance. This can be inferred from the unweighted comparison (Fig. 2B) which shows an increase in dissimilarity between each pair of methods (see additional details in Supplementary and Fig. S2).

These comparisons only elucidate the similarity between a pair of methods. To determine which tool most accurately recapitulates the reference sequences in the samples, we applied the same pipeline step to process the mock data sets (mock4, mock12, and mock16) and compared the predicted representative sequences with the true sequences and their distribution. The results (Fig. 2C and D) show that the predicted sequence distributions are overall different from the expected ones. The variation across data sets indicates that the data sets themselves play a big role in method performance. We note that there is no method that outperforms the rest in all data sets (see Supplementary for an extended discussion). Based on being among the top performers on the mock data sets, their *de novo* error-correcting nature, and previous independent evaluation (60), DADA2 and Deblur appear to be the most reliable. This is because the open-reference and *de novo* clustering methods return a much larger number of OTUs compared to the other pipelines and would affect the accuracy of the NI step if stringent filtering is not performed. Overall, since DADA2 as compared to Deblur displays better performance on all the mock data sets on the weighted UniFrac metric, we set this tool as the default for the DC step of the pipeline. However, if comparison across studies that have sequenced different 16S regions is required, closed- and open-reference might be a better option.

After the denoising, the sequences are subject to chimera checking (CC). The MiCoNE pipeline supports two different CC methods: “uchime-denovo” (51) and “remove bimera” (42). We did not notice any notable difference between the two methods (Fig. S3), implying that they identify and remove mostly the same set of sequences as chimeras. Since the remove bimera method was originally developed in conjunction with DADA2, we use this method as the default. The DC step, thus, results in a reduced set of unique sequences, which will be referred to as representative sequences in the subsequent steps.

### TA: Taxonomy databases vary widely in taxonomy assignments beyond order level

Taxonomy databases are used to assign taxonomic identities to the representative sequences obtained after the DC step. The three 16S taxonomic reference databases used in this study are SILVA (61), Greengenes (GG) (57), and National Center for Biotechnology Information (NCBI) RefSeq (62) (see Methods). These databases vary substantially in terms of taxonomy hierarchies, including species names and phylogenetic relationships (63). Assignment using a particular database also requires a query tool. We used the “Naive Bayes” classifier from QIIME2 for the GG and SILVA databases and the “BLAST” tool (included as a QIIME2 plugin) for the NCBI database. These tools have been well quantified and optimized (44); hence, we made use of the default parameters in our analyses.

**Fig 3:**
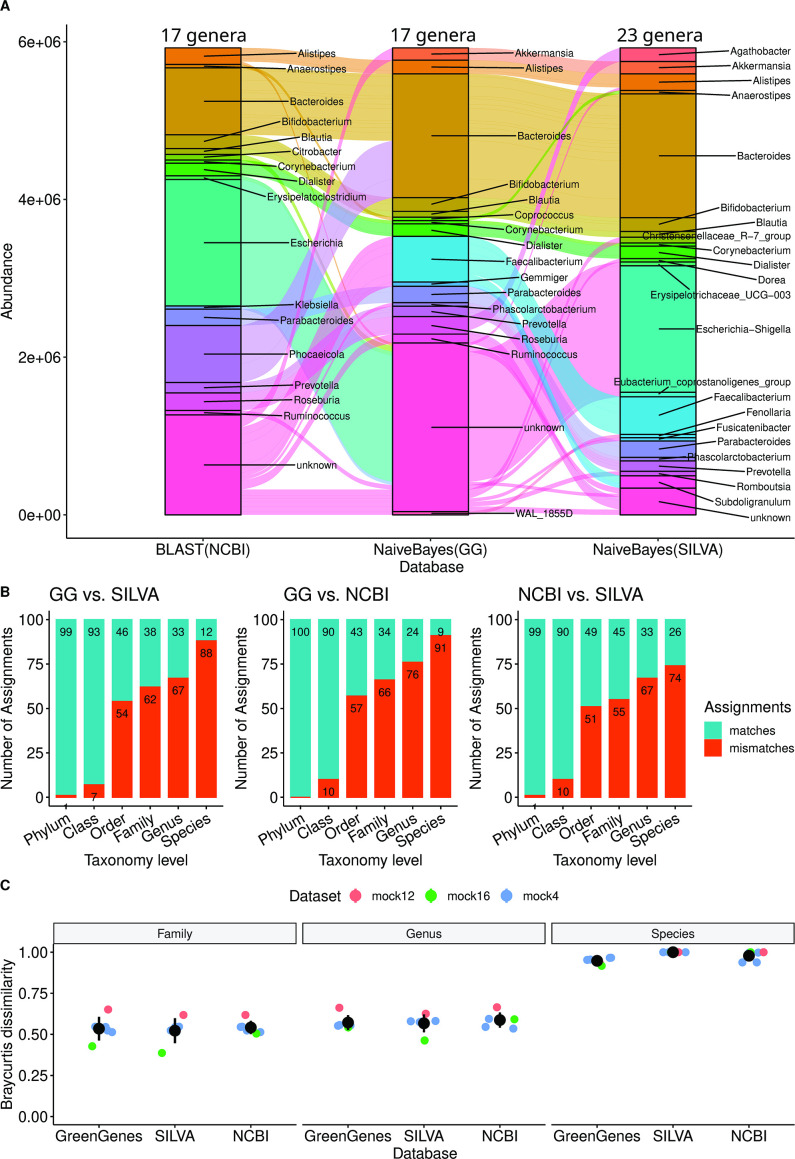
Taxonomic reference databases vary in terms of their taxonomy assignments below the order level. (**A**) The taxonomic assignments of the top 50 representative sequences using the three different reference databases. This result illustrates how the same sequences are assigned to different genera under different databases. A significant portion of the representative sequences is assigned to an “unknown” genus in two of three databases (GreenGenes and NCBI). The number of assigned genera for each database is displayed at the top of each column. (**B**) The number of representative sequences assigned to the same taxonomic label when using different reference databases (for the top 100 representative sequences). The mismatches are fewer at higher taxonomic levels but, even at the order level there exists greater than 51% of mismatches, demonstrating the poor agreement in taxonomic labels assigned by the different databases. The data used for the analysis in (**A and B**) were samples (healthy and ASD) from the FMT data set. (**C**) The Bray-Curtis dissimilarity between the predicted taxonomy profile and expected taxonomy profile in the mock data sets shows that there is no singular best choice of database for every data set, as all the databases show similar performances. The GreenGene database and the Naive Bayes classifier are chosen as the defaults for the TA step of MiCoNE due to their popularity. The data sets used for the analysis in (**C**) were the mock data sets from mockrobiota.

The representative sequences obtained using the default settings of the DC step were used for taxonomic assignment using the three reference databases. Fig. 3A depicts a flow diagram that shows how the top 50 representative sequences (sorted by abundance) are assigned a Genus according to the three databases. The different databases lead to assignments that qualitatively display similar distributions. However, the assigned Genus compositions also display clear differences, as does the percentage of unassigned representative sequences (pink). Some of the differences in Genus composition have a clear explanation, for example, abundant Genera such as *Bacteroides* and *Escherichia* are assigned to different representative sequences. The large percentage of unassigned sequences is due to the large fraction of the representative sequences assigned to an “unknown” Genus during the assignment process (see Methods).

After the assignment, we performed a pairwise comparison of the similarity between the top 100 assignments (by abundance) from different databases at every taxonomic level (Fig. 3B). The comparisons of the assignments below the order level (Family, Genus, and Species) show less than 45% similarity between any pair of databases. This implies that the assignments from each reference database are fairly unique. The comparison of all assigned genera (Fig. S4), instead of just the top 100, contains a higher percentage of mismatches. This suggests that, comparatively, the most abundant sequences are more consistently matched to the same taxonomies, at least for the data set tested in the current analysis.

To obtain an absolute measure of the accuracy of the taxonomic assignments, we used the representative sequences from the DC step for mock data sets as the query sequences and the expected taxonomic composition as the standard to compare against. We used the Bray-Curtis distance metric (64) to calculate the distance between the predicted and expected taxonomic distribution (Fig. 3C). We find that none of the databases perform better than the others in absolute terms and that the dissimilarity with the expected composition is high (> 0.5 for Family and Genus and > 0.9 for Species), indicating that all the databases have some limitations when trying to recapture the expected taxonomic composition.

Since no database performs better than others against mock data sets, the choice of which database to use could be driven by other reasons (see Supplementary discussion). One reason to choose a particular database could be the frequency of updates and the potential for future growth. Both GG, due to its frequent use in the literature (63), and NCBI, due to its regular revision and maintenance, could be good choices for TA. In our default pipeline, we choose GG as the default method.

The TA step results in a taxonomic counts table that is used as input to the subsequent steps of the pipeline. Note that the count tables at different levels can be obtained through aggregation; for example, Genus count tables were obtained by summing up the counts of the lower taxonomy levels (Species and OTU) that map to the same higher taxonomy level entity.

### NI: Different network inference methods drastically affect edge-density and connectivity

The ten NI methods we used in this step fall into two groups: the first set of methods (Pearson, Spearman, SparCC (38, 46) and propr (65)) infers pairwise correlations, while the second set (SpiecEasi (47), FlashWeave (48), COZINE (66), HARMONIES (67), SPRING (68), and mLDM (69)) infers direct associations. In general, when we refer to co-occurrences, we include the associations from both correlation-based methods and direct association-based methods. On the other hand, when mentioning correlations, we will exclusively refer to edges inferred by the correlation methods. Note that while Pearson and Spearman methods are included in the pipeline for completeness, they tend to generate a large number of spurious edges as they are not intended for compositional data sets. Thus, they are not included in subsequent quantitative analyses.

**Fig 4:**
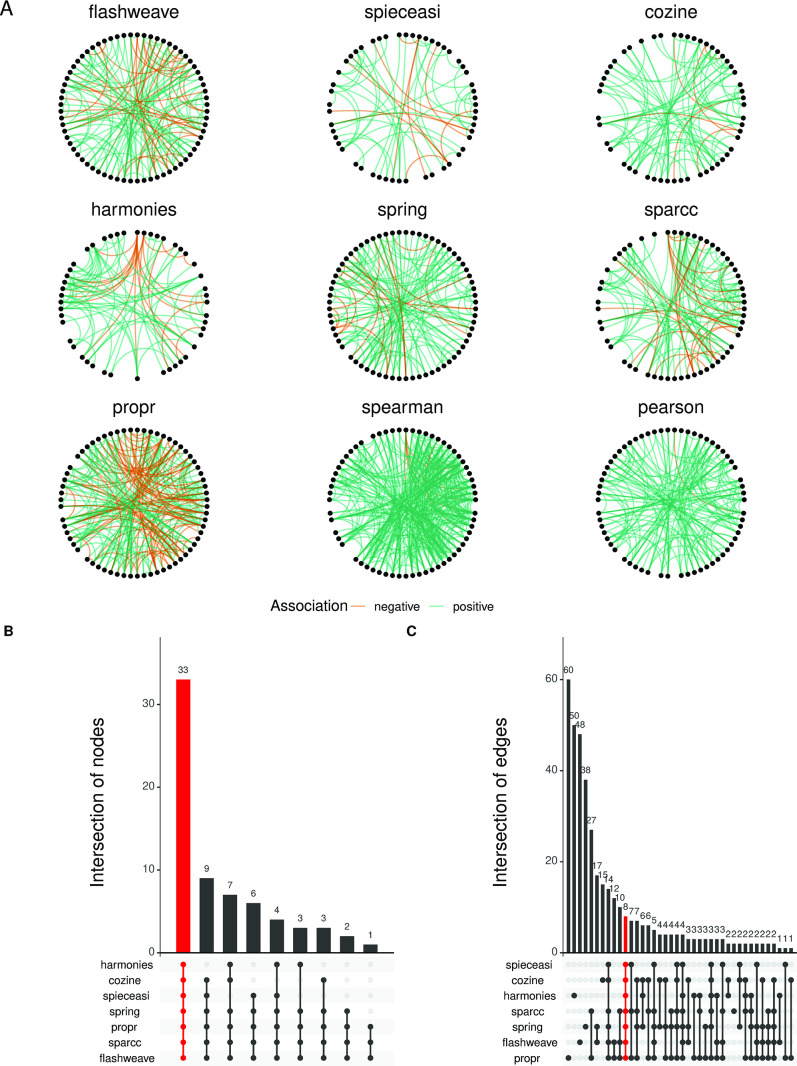
Networks generated using different network inference methods show notable differences in terms of edge-density and connectivity. (**A**) The nine different networks generated by the different network inference methods (excluding mLDM). The nodes for each network (representing taxa) are arranged in the same positions in a circular layout, and the differences in the connections can be directly visualized and compared. The green links are positive associations, and the orange links represent negative associations. The networks look dissimilar and vary widely in terms of connectivity, and it is notable that the correlation-based methods generally produce networks with higher edge-densities. A threshold of 0.3 was set for the correlation-based methods (sparcc, propr, spearman, and pearson), and a threshold of 0.01 was set for the direct association methods (flashweave, spieceasi, cozine, harmonies, and spring). (**B**) The node overlap Upset plot indicates that all the networks have a large proportion of common nodes involved in connections (33 out of 68). Conversely (**C**), the edge overlap Upset plot shows that a very small fraction of these connections are actually shared (8 out of 202). The data used in this analysis were the healthy stool samples from the FMT data set. mLDM is not shown in the comparisons because the algorithm failed to converge for the particular network combination used here (default setting of the MiCoNE pipeline).

Filtered (see OP step in Methods) genus-level counts table obtained using the default settings in the previous steps were used as input for the different NI algorithms (Fig. 4). Even from a visual inspection (Fig. 4A), one can see that the different networks differ vastly in their edge-density and connectivity, with common edges often displaying inverted signs.

To quantify the differences between the networks, we analyzed the distribution of common nodes and edges (Fig. 4B and C) using UpSet plots (70). The node intersection analysis shows that the networks have 33 out of 68 total unique nodes in common and that no network possesses a unique node (only nodes with connections are included). Edge intersections in contrast show that only 8 edges (out of 202 total unique edges) are in common between all the methods, and each network has many unique edges. These results indicate a substantial rewiring of connections in different inferred networks and prompted us to identify associations robust across methods, through consensus algorithms.

### NI: The scaled-sum consensus method shows high precision in benchmark data sets

Inspired by previous approaches (71, 72), we developed two methods that take into consideration the evidence offered by each NI algorithm and generate a consensus network that contains the common edges among the inferred networks.

Both of our approaches—simple voting (SV) and scaled-sum (SS)—combine appropriately filtered networks inferred from correlation-based and direct association methods (see Methods). We chose the SS method as the pipeline default since this method takes into account the weights of the associations in the determination of the final consensus. The pipeline enables the selection of any subset of methods for the consensus calculation. Currently, by default, all direct association methods (SpiecEasi, COZINE, HARMONIES, SPRING, mLDM, and FlashWeave) are used, together with SparCC and propr.

**Fig 5:**
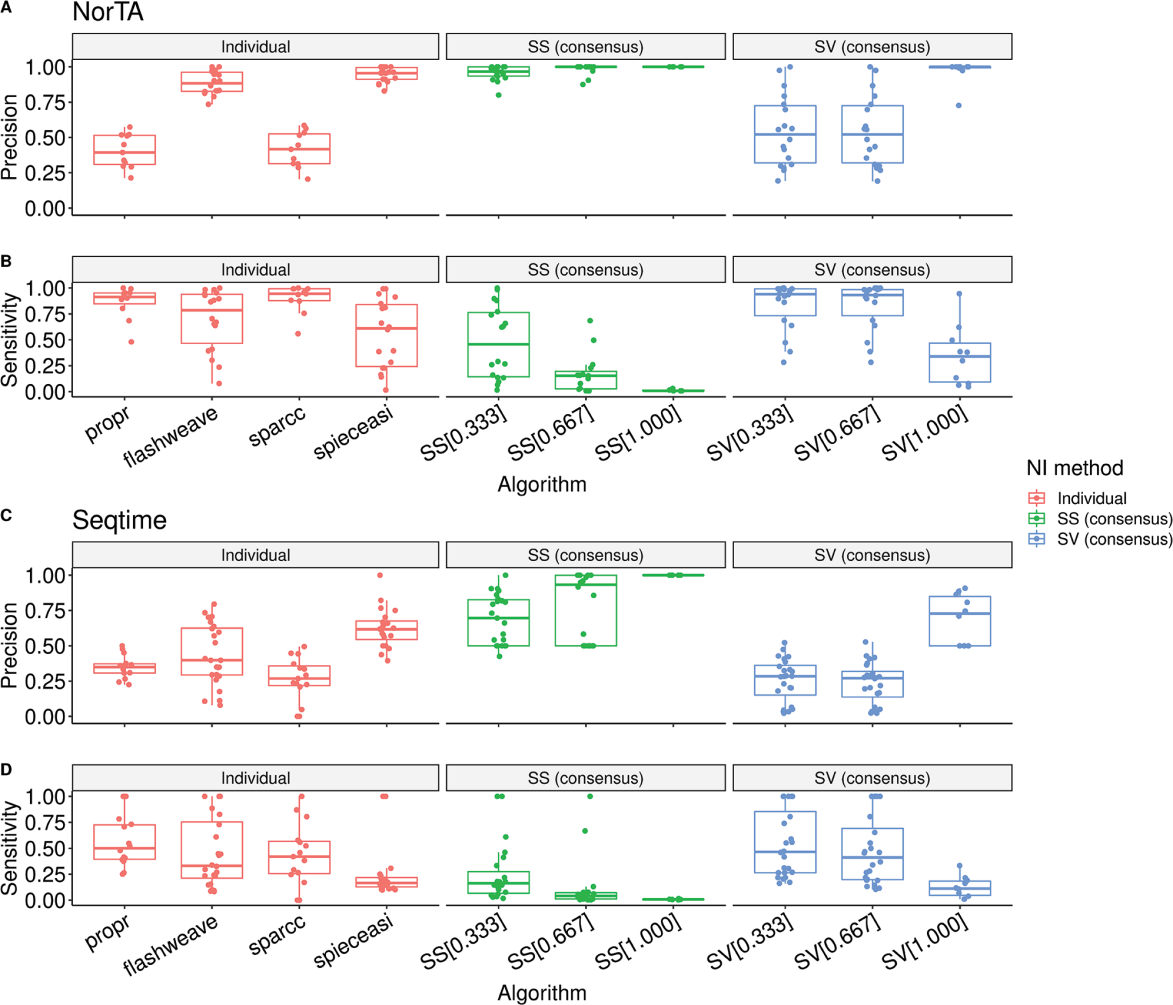
The associations generated by the scaled-sum consensus method show high precision in benchmarks using synthetic data sets. The different points on the box plot show the precision (**A and C**) and sensitivity (**B and D**) of co-occurrence networks generated through individual network inference methods and consensus network construction approaches. Precision and sensitivity are estimated based on the comparisons with two sets of synthetic benchmark data sets (“NorTA” and “seqtime,” see Methods). The independent algorithms chosen for the comparison are the two best-performing correlation-based (propr, sparcc) and direct-association-based (spieceasi, flashweave) methods. For consensus network inference, we used the scaled-sum (SS) and simple voting (SV) methods. A weight threshold of 0.1 and a *P*-value threshold of 0.05 were applied to each network before the calculation of precision and sensitivity. The purpose behind the construction of the consensus algorithms is to capture true associations in the data through the removal of associations that have a lower probability of being present in the networks inferred by different inference algorithms. Therefore, an increase in precision is followed by a decrease in sensitivity. The SS consensus method consistently obtained the overall best precision for θ ≥ 0.333 on both benchmark data sets. Among all the individual network inference methods, spieceasi shows the best average precision. When using the presence of edges in all inferred networks as a requirement (θ = 1.000), the simple voting method also outperforms spieceasi on average precision. Therefore, we set the scaled-sum consensus method with θ = 0.333 as the default tool for consensus network inference since this option provides a good balance of precision and sensitivity (see also Fig. S5 and S6). The correlation-based methods (propr and SparCC) and the simple-voting consensus method return networks with higher sensitivities.

Similar to what was done for the previous steps of the pipeline analysis, and in analogy with previous estimations of NI accuracy (47, 53), we evaluated the NI algorithms and the final consensus network using synthetic interaction data. For this purpose, we generated synthetic interaction data using the “NorTA” (47) and “seqtime” (73) methods (see Methods). For each method, an OTU counts table was generated based on the selected parameters and abundance distributions. This counts table was used as the input to the MiCoNE pipeline to generate predicted associations. The interaction network used to generate the counts table was used as the source of true interactions to calculate the precision and sensitivity (Fig. 5) of the consensus algorithms. These values are also compared with the precision and sensitivity of four individual NI methods (two correlation-based methods, i.e., propr and FlashWeave, and two direct association methods, i.e., SparCC and SpiecEasi), which were selected based on their high precision (see Fig. S5 and S6). As shown in Fig. 5, the consensus algorithm, especially the SS method, captures true associations with high precision (through the removal of edges that are either not present in most of the inference methods or whose association strength is low across methods). Although this increase in precision is associated with a drop in sensitivity (as the consensus parameter θ increases), the consensus networks provide valuable and practically helpful results, in the form of a short list of high-confidence associations. Overall, the SS method for θ = 1.000 performs the best (precision = 1.000 for both NorTA and seqtime). Fig. S5 and S6 show the precision and sensitivity values of all NI and consensus algorithms for each interaction network in the synthetic data sets. The SS method for θ = 0.333 (default option in the pipeline) shows a high precision (0.956 with NorTA; 0.688 with seqtime) without displaying a significant reduction in sensitivity (Fig. 5; Fig. S5 and S6 ). However, if higher precision is required, θ > 0.5 can be considered.

### Impact of different pipeline steps on co-occurrence networks

**Fig 6:**
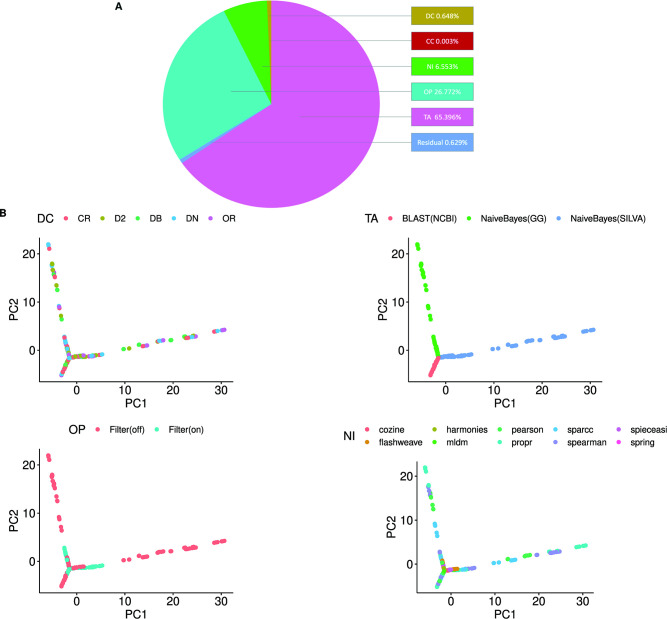
The choice of reference database has the largest impact on network variance. (**A**) The percentage of variance in the networks (generated from the FMT data set) contributed by the denoising and clustering (DC), chimera checking (CC), taxonomy assignment (TA), OTU processing (OP), and network inference (NI) steps of the pipeline calculated using ANOVA on a linear model (see Methods). A weight threshold of 0.1 and a *P*-value threshold of 0.05 were applied to each network before the analysis. The taxonomy database contributes most to the variance between the networks (65.4%) followed by the filtering of the counts matrix (26.8%) in the OP step. The variation due to the NI, DC, and CC steps is much smaller in comparison (6.553%, 0.648%, and 0.003%, respectively). The negligible fraction labeled as the residual is an artifact that arises when multiple steps are changed at the same time. (**B**) All the inferred networks generated from various combinations of tools are shown as points on a PCA plot. Each point on the PCA plot represents a network inferred using different combinations of tools and parameters that are available in the MiCoNE pipeline. The color of the points corresponds to the tools used at each step of the pipeline (DC, TA, OP, and NI). The points on the PCA plot can be grouped based on the TA step, but the extent of this separation decreases when the filtering is turned on in the OP step, confirming that the variability in the networks decreased upon filtering out the taxonomic entities at low abundance. Some algorithms, especially the direct association methods, at the NI step can also be seen to generate networks that are less variable compared to the others. The DC step does not seem to have any correlation with the variation in the networks on the PCA plot.

In order to analyze the effect of different processing methods on the inferred co-occurrence networks (before consensus estimation), we generated networks using all possible combinations of methods and quantified the variability due to each choice (Fig. 6A). This was achieved by building a linear model of the edges of the network as a function of the various steps in the pipeline workflow (see Methods). Fig. 6A shows the percentage of total variation among the co-occurrence networks due to the different steps of the pipeline. The TA step, or more specifically the choice of 16S reference database, contributes the most (65.4%) to the variation in the networks, followed by the OP step (26.8%). This result highlights the importance of the TA step in the 16S data analysis workflow, implying that a change in the reference database will result in drastically different inferred networks. This is likely due to the differential assignment of representative sequences to taxonomic entities (Fig. 3; Fig. S4), which drastically alter the nodes and, hence, the underlying network topology.

The effects of the different steps of the pipeline on the inferred networks can be visualized through dimensionality reduction. The PCA in Fig. 6B shows all the above networks, colored by the tools used in the DC, TA, OP, and NI steps in each subfigure. The major effect of the TA step choice, shown before in Fig. 6A, is also reflected in the PCA plot, where networks segregate based on the database used (Fig. 6B; Fig. S1). Additionally, the plot also shows that the variation between the networks decreases when the low abundance OTUs are removed from the network. It is also evident that, in the NI step, some networks, especially those inferred using the direct association NI methods, are much closer in the PCA plot regardless of the reference database used. The network variance analysis performed on the supplementary data set (74) (stool samples from radiation-exposed bank vole) shown in Fig. S8 supports these observations, implying that these findings are fairly consistent across different data sets. These results suggest that the most important criterion for accurate comparative analysis of co-occurrence networks is the taxonomy reference database followed by the level of filtering of the taxonomy tables and the NI algorithm used.

### The default pipeline

The systematic analyses in the previous sections illustrate that the choice of tools and parameters can have a big impact on the final consensus co-occurrence network. However, the mock communities and synthetic data provide an opportunity to select combinations of tools that yield the most accurate and robust results. As highlighted in the above sections for individual steps, we propose a set of tools and parameters as the defaults for the pipeline (Table 1).

**TABLE 1:** Tools used in the MiCoNE pipeline^*a*^

Workflow step	Module/condition	Tool/parameter	References/value
Denoising and clustering	Denoise and cluster	Closed reference	[51]
		Open reference	[51]
		*De novo*	[51]
		DADA2	[42]
		Deblur	[43, 51]
	Chimera checking	Uchime-*de novo*	[51]
		Remove bimera	[42]
Taxonomy assignment	Query tool	Blast	[44]
		Naive bayes classifier	[44]
	Database	Greengenes 13 8	[57]
		SILVA 138	[61]
		NCBI RefSeq (Oct 2021)	[62]
OTU processing	Filter(off)	Prevalence threshold	2/*n* samples
		Abundance threshold	0.001
		Observation sum threshold	10
	Filter(on)	Prevalence threshold	0.05
		Abundance threshold	0.01
		Observation sum threshold	100
Network inference	Bootstrapping	Fastspar bootstraps v1.0	[46]
		fastspar pvalues v1.0	[46]
	Direct association	SpiecEasi v1.1.2	[47]
		FlashWeave.jl v0.18.1	[48]
		COZINE v1.0	[66]
		HARMONIES v1.0	[67]
		SPRING v1.0.4	[68]
		mLDM v1.1	[69]
	Correlation-based	SparCC (FastSpar v1.0)	[46]
		Pearson	-
		Spearman	-
		propr v2.1.2	[65]
	Consensus algorithm	Scaled-sum	0.333
		Simple voting	1.000

^*a*^ The tools highlighted in gray are the defaults for the pipeline that are recommended based on the benchmarks with the mock and synthetic data sets. The consensus algorithm in the network inference (NI) step incorporates all the modules (bootstrapping, direct association, and correlation-based) to generate the consensus network.

**Fig 7:**
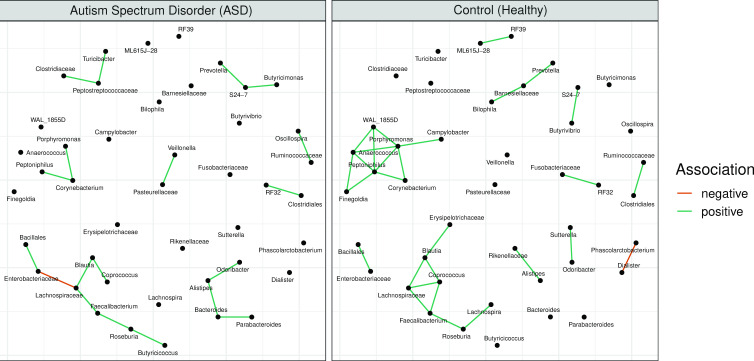
Comparison of networks generated from the control and ASD samples of the FMT data set using the MiCoNE pipeline. The networks for the ASD (left) and control (right) samples were generated using the default tools and parameters recommended by the MiCoNE pipeline as described in Table 1. There are 22 unique links in the network for control samples, 12 unique links in the network for ASD subjects, and 7 edges in common between both networks. The changes in these connections can serve as potential starting points for further experimental validations or literature surveys.

Fig. 7 shows the co-occurrence networks inferred for the healthy subjects (control) and subjects with autism-specific disorder (ASD) in the FMT study (55) (constructed using the default tools and parameters from Table 1). This figure demonstrates a typical use case of comparative analysis of networks using the MiCoNE pipeline. As a consequence of using the consensus network algorithm, the final co-occurrence networks are sparse and can be visually compared and examined.

The analysis of the rewiring of associations in the ASD samples with respect to the control provides a guide for the identification of key genera that could be linked to dysbiosis. We observed 22 unique links in the network for control samples, 12 unique links in the network for ASD subjects, and 7 edges in common between the two networks. Although these unique associations do not imply actual interactions, they can still serve as potential starting points for literature surveys and further experimental exploration of mechanistic processes underlying dysbiosis. For example, *Prevotella* and *Porphyromonas*, genera previously implicated in ASD (55, 75) and cognitive impairment (76), display modified connectivity in our network, suggesting that the observed associations may be relevant for understanding the role of these bacteria in disease. Additional visualization and comparison of networks can be performed using the MIND (54).

Fig. S7 shows a sensitivity analysis in which we compared the default network against networks generated by altering one of the steps of the pipeline relative to the default. This result, both visually (Fig. S7A) and quantitatively (Fig. S7B), suggests that the most significant changes occur when the OP or TA steps are changed from the default value.

## DISCUSSION

### Why MiCoNE?

A myriad of tools and methods have been developed for different parts of the workflow for inference of co-occurrence networks from 16S rRNA data. Our analyses have shown that networks generated using different combinations of tools and approaches can be substantially different from each other, highlighting the need for a clear evaluation of the source of variability and tools that provide the most robust and accurate results. Our newly developed software, MiCoNE, is a customizable pipeline for the inference of co-occurrence networks from 16S rRNA data that enable users to compare networks generated by multiple possible combinations of tools and parameters. Importantly, in addition to revisiting the test cases presented in this work, users will be able to explore the effect of various tool combinations on their own data sets of interest. The MiCoNE pipeline has been built in a modular fashion; its plug-and-play architecture enables users to add new tools and steps, either using existing packages that have not been examined in the present work or those developed in the future. The MiCoNE Python package provides functions and methods to perform a detailed analysis of the count matrices and the co-occurrence networks. The inferred networks are exported to a custom JSON format (see Supplementary) by default but can also be exported to Cytoscape (77), GML (78), and many other popular formats via the Python package.

While several tools/workflows such as QIIME2 (51) and NetCoMi (79) can be used to generate co-occurrence networks from 16S sequencing data, no single tool exists that integrates the complete process of inferring microbial interaction networks from 16S sequencing reads. MiCoNE is unique as it offers this functionality packaged in a workflow that can be run locally, on a computer cluster, or in the cloud.

### The default pipeline and recommended tools

Through MiCoNE, in addition to transparently revealing the dependence of co-occurrence networks on tool and parameter choices (see discussion in Supplementary text for details on the DC, TA, and OP steps), we have taken advantage of our spectrum of computational options and the availability of mock and synthetic data sets to suggest a default standard setting that streamlines comparisons across data sets. Additionally, we have developed a consensus approach that can reliably generate fairly robust networks across multiple tool choices. Even if, in the current analysis, we have shown the relevance of our approach to two very different types of microbiome data sets (human and vole stool), it is important to remember that there is no universal standard for microbial interaction data, and our conclusions are based on the specific data sets used in our analysis. While our analysis is based on several mock and synthetic data sets that cover a diverse range of abundance distributions and network topologies, data sets with drastically different distributions may require a re-assessment of the best settings. However, the MiCoNE pipeline provides a platform for easy evaluation of accuracy, variance, and other properties at each workflow step for any other data set of interest.

The networks generated by different network inference methods show considerable differences in edge-density and connectivity, partially due to the underlying assumptions regarding sparsity, distribution, and compositionality. To address this issue, we have developed two consensus algorithms (simple voting and SS method) that generate networks whose links have evidence based on multiple inference algorithms.

We find that the SS method performs the best on synthetic data sets and is, therefore, chosen as the default for the NI step of the pipeline. Notably, the consensus network displays a higher precision and returns a concise list of robust associations representing a valuable set for experimental validation follow-up.

### Future directions

Future work building upon our current results could enhance the network inference process in multiple ways. The current analyses make use of a FMT data set with control and ASD samples, stool samples from radiation-exposed bank vole, three mock community data sets, and several data sets generated by two synthetic interaction methods. Incorporating data sets from a broad spectrum of biomes with varying microbial distributions into MiCoNE will likely increase the robustness and generalizability of the results from these analyses.

The network analyses in this study are primarily at the Genus level, wherein the lowest resolution of a node is a Genus, and if an entity cannot be resolved to the Genus level, the next taxonomic level is used (e.g., Family). Consequently, two entities belonging to the same lineage where one entity is resolved to the Genus level and another is resolved to the Family level are treated as two different nodes in the network. Thus, developing an overlap metric to compare nodes with shared lineages within and across networks could enable more biologically and phylogenetically relevant comparisons.

In thinking about the possible biological interpretations of the co-occurrence network computed by us and others, it is important to remember that there is no solid basis for assuming that these networks carry information about physical or metabolic interactions (80, 81). Comparing co-occurrence networks and directly measured interactions remains a major unresolved challenge (82, 83), which needs to be investigated further. Understanding this connection will be beneficial for predicting interactions in systems where direct interaction measurements cannot be taken. Further, benchmarking of co-occurrence networks could also be pursued through the use of literature-based interactions (1) or biological benchmark interaction data (84). Additionally, MiCoNE could be extended to enable the processing of metagenomics sequencing data, facilitating the analysis of a much larger and diverse range of data sets and domains of life.

Although in the current analysis, we have only used default parameter values recommended by the tool creators, the MiCoNE pipeline could be used in the future to explore any combinations of parameters and optimize these values for improved network inference. Overall, there likely is no “best method” for the various steps of 16S data analysis, and hence, MiCoNE is intended to help researchers to identify the methods and algorithms that are most suitable for their data sets in an easy-to-use and reproducible manner.

We envision that MiCoNE, and its underlying tools and databases, will be increasingly useful for building large comparative analyses across studies. It enables rapid, configurable, and reproducible inference of microbial networks and furthers the formulation of hypotheses about the role of these interactions on community composition and stability. These comparative analyses will require coupled network analysis and visualization tools (such as MIND (54)) and need systematic access to data sets, shared in accordance with FAIR standards (81).

## MATERIALS AND METHODS

### 16S rRNA sequencing data sets

This study utilized two types of 16S rRNA sequencing data sets: biological data sets and mock/synthetic data sets. Biological data sets are collections of sequencing reads obtained from naturally occurring microbial community samples. The current analysis used stool samples from the FMT study of autism (55) as the biological data set. This data set was chosen because the sequences were easily accessible on Qiita (50, 85) and optimally pre-processed according to the Earth Microbiome Project (EMP) (2) protocol, allowing them to be used directly as input to the MiCoNE pipeline. The study was composed of multiple sequencing runs. The runs that contained paired-end reads (run 2 (10M reads), run 3 (750K reads), and run 4 (16M reads)), were downloaded from Qiita (50, 85) (study ID 10532) and used as input sequences for the MiCoNE pipeline. Sequences from both control (212 samples including neurotypical and donors) and ASD (126 samples) patients were included in the analyses. All the network analyses in the study, unless explicitly mentioned, were performed on the control and ASD samples in the FMT study. The mock community 16S data sets are experimental sequencing data obtained for artificially assembled collections of DNA of species in known proportions. The mock data sets used for this study, obtained from mockrobiota (56), are labeled mock4, mock12, and mock16. The mock4 community is composed of 21 bacterial strains. Two replicate samples from mock4 contain all species in equal abundances, and two additional replicate samples contain the same species in unequal abundances. The mock12 community is composed of 27 bacterial strains that include closely related taxa with some pairs having only one to two nucleotide differences from one another. The mock16 community is composed of 49 bacteria and 10 Archaea, all represented in equal amounts. In addition to these data sets, we have utilized a data set containing stool samples from radiation-exposed bank vole (74) (Qiita study ID 13114) for supplementary analyses.

### MiCoNE

The flowchart describing the workflow of MiCoNE, our complete 16S data-analysis pipeline, is shown in Fig. 1. The pipeline integrates many publicly available tools as well as custom R or Python modules and scripts to extract co-occurrence associations from 16S sequence data. Each of these tools corresponds to a distinct module that recapitulates the relevant analyses. All such individual modules are available as part of the MiCoNE package. The inputs to the pipeline by default are raw untrimmed 16S rRNA sequence reads, but the software can be alternatively configured to use trimmed sequences, OTU tables and other types of intermediate data (see documentation). The configuration and modular nature of the MiCoNE package enables users to start and end the pipeline at any point in the workflow and run parts of the pipeline in isolation. The pipeline supports both paired-end and single-end reads and additionally supports independently processing reads from multiple runs and merging the OTU tables in the DC step. The final output of the pipeline is the inferred network of co-occurrence relationships among the microbes present in the samples.

The MiCoNE pipeline provides both a Python API together with a command-line interface and only uses a single configuration file (nextflow.config) to encode the configuration parameters. The MiCoNE Python API provides several OTU table and network-related functions and methods, enabling detailed comparison of counts tables and inferred networks if desired. Exploring the effects of these combinations of methods on the resultant networks is difficult and inconvenient since different tools differ in their input and output formats and require interconversions between the various formats. The pipeline facilitates this comparative exploration by providing a variety of modules for interconversion between various formats and allowing for easy incorporation of new tools as modules. It also contains helper functions that can help in parsing taxonomies and communicate with the NCBI taxonomy database to query taxonomy by name or taxonomic IDs. The configuration file along with the run file (main.nf) lists the inputs, output, and the steps to be performed during runtime along with the parameters to be used (if different from defaults) for the various steps. The default settings of the pipeline are shown in Table 1 (with default parameter values shown in Tables S2–S6 at https://github.com/segrelab/MiCoNE-pipeline-paper). Since the entire pipeline run is stored in the form of a text file (the configuration file), subsequent runs are highly reproducible and changes can be easily tracked using version control. The pipeline makes use of the nextflow workflow manager (86) under the hood, making it readily usable on the local machine, cluster, or cloud with minimal configuration change. It also allows for automatic parallelization of all possible processes, both within and across samples. The pipeline is designed to be modular: each tool or method is organized into modules that can be easily modified or replaced. This modular architecture simplifies the process of adding new tools (refer to the modules section in the MiCoNE documentation). The main components of the pipeline are detailed in the subsequent sections.

### Sequence processing (SP)

This module deals with processing the raw multiplexed 16S sequence data into demultiplexed, quality-controlled, trimmed sequences. It consists of the demultiplexing and trimming processes. The demultiplexing process deals with separating the multiplexed sequences into individual samples based on barcodes. The trimming process handles the quality control steps such as trimming adapters and low-quality nucleotide stretches from the sequences. The parameters and tools in this process are fixed and are not available for user customization. The various tools used for the processes were adapted from QIIME2 v2021.8.0 (51). The list of tools used in this step, along with their modules and references, is provided in Table 1.

### Denoising and clustering (DC)

This module deals with processing the quality-controlled, trimmed 16S sequence data into OTU or ESV count tables. It consists of the following processes: denoising (or clustering) and CC. The denoise/cluster process handles the conversion of the demultiplexed, trimmed sequences into OTU or ESV count tables (some methods, like closed reference and open reference clustering, make use of a taxonomy reference database for clustering). The chimera checking process handles the removal of chimeric sequences created during the polymerase chain reaction step. The output of this module is a matrix of counts that describes the number of reads of a particular OTU or ESV (rows of the matrix) present in each sample (columns of the matrix). The options currently available in the pipeline for DC are open reference clustering, closed reference clustering, and *de novo* clustering methods from the vsearch plugin of QIIME2 v2021.8.0(51) and denoising methods from DADA2 v1.14 (42) (from the DADA2 R package) and Deblur v1.1.0 (43) (from the deblur plugin of QIIME2). The quality filtering and CC tools are derived from those used in QIIME2 v2021.8.0 (uchime-*de novo* method) and DADA2 (remove bimera method). The list of tools used in this step, along with their modules and references, is provided in Table 1.

For the UniFrac analysis in Fig. 2, we had set a count threshold of 10, such that if the count of the representative sequences in a particular sample is less than the threshold, it is omitted from the analysis. Additionally, for Fig. 2C and D, the expected sequences from the mock communities were trimmed to the V4 region before being subject to UniFrac analyses.

### Taxonomy assignment (TA)

This module deals with assigning taxonomies to the representative sequences (OTUs or ESVs). In order to assign taxonomies to a particular sequence, a taxonomy database and a query tool are necessary. The taxonomy database contains a collection of 16S sequences of microorganisms, and the query tool allows one to compare a sequence of interest to all the sequences in the database to identify the best matches. Finally, a consensus method is used to identify the most probable match from the list of best matches. The pipeline incorporates GreenGenes (GG) 13_8 (57) (99% identity), SILVA 138 (61) (99% identity), and the NCBI (16S RefSeq as of October 2021) (62) databases for TA. SILVA and GG are two popular 16S databases used for taxonomy identification, and the NCBI RefSeq nucleotide database contains 16S rRNA sequences as a part of two BioProjects—33175 and 33317. The three databases vastly differ in terms of their last update status—GG was last updated in May 2013, SILVA was last updated in August 2020 at the time of writing, and NCBI is updated regularly as new sequences are curated. These databases were downloaded and built using the RESCRIPt QIIME2 plugin (87). The Naive Bayes classifier and the NCBI blast used as the query tools in this study were from the QIIME2 package, and the parameters used were the defaults of the package. The consensus algorithm used is the default method used by the classifiers in QIIME2. During the assignment, a representative sequence might be assigned an “unknown” Genus for one of two reasons: the first is if the taxonomy identifier associated with the sequence in the database did not contain a given Genus; the second, more likely reason, is that the database contains multiple sequences that are very similar to the query (representative) sequence and the consensus algorithm (from QIIME2) is unable to assign one particular Genus at the required confidence. The assignments in SILVA were originally substantially different from the other two databases ( 40% mismatch) even at the Phylum level. However, this was corrected via minor adjustments to the taxonomic names, such as changing Bacteroidota to Bacteroidetes in the SILVA Phylum assignments. The full list of changes can be found in script *figure4ab_data.py* in the data and scripts repository. The list of tools used in this step, along with their modules and references, is provided in Table 1.

### OTU and ESV processing

This module deals with normalization, filtering, forking, grouping, and applying transformations to the OTU or ESV counts matrix. Normalization of the count matrix involves converting the count matrix of read counts into a count matrix containing relative abundances. The module also supports rarefaction, which is a normalization technique used to overcome the bias that might arise due to variable sampling depth in different samples. This is performed by subsampling of the matrix to a specified rarefaction depth (45) in order to obtain samples with equal library sizes. However, due to the potential biases and false positives (88, 89) that might arise during the process, the rarefaction module is disabled by default and can be enabled in the configuration if needed. Hence, although the pipeline supports rarefaction, it is turned off by default. In addition to rarefaction, the MiCoNE pipeline also supports total sum scaling and the centered log ratio transformation (from the speiceasi R package). However, since most of the NI methods perform normalization and other transformation operations on the counts matrix as a part of their workflow, the analyses reported in the paper do not explicitly normalize the counts matrices. Filtering is performed to remove samples or features (OTUs or ESVs) from the counts matrix that are sparse. By default, when the OP module is “on,” the samples are filtered out if the total reads in a sample are less than 500 and features are filtered out if the relative abundance is less than 1%; prevalence (percentage of samples containing feature) is less than 5% and count sum across all the samples is less than 100. When the OP module is “off,” the filtering is still performed, but threshold parameters are much more relaxed. The parameters used are given in Table 1. The forking operation splits the count matrix into multiple matrices based on sample metadata column; this is useful, for example, to compare case versus control. The group operation transforms the OTU or ESV count matrix into a taxonomic count matrix at the requested level by adding up counts that map to the same taxonomy and is carried out at the end of the OP step. Finally, transformations are performed in order to correct for and overcome the compositional bias that is inherent in the counts matrix (in the analysis performed in the study, these were disabled and directly handled by the NI algorithm). All the modules in this step were implemented using functions from the biom-format Python package (90).

### Network inference (NI)

This module deals with the inference of co-occurrence associations from the processed taxonomic counts matrix. The input count matrices are collapsed to the Genus level (or any other required taxonomy level) using the group module at the OP step. These collapsed matrices are used as input to the NI methods to produce association matrices at the appropriate taxonomy level. These associations can be represented as a network, with nodes representing the taxonomies of the microorganisms and edges representing the associations between them.

The pipeline includes four methods for pairwise correlation metrics and six methods for direct association metrics (refer to Table 1). Pairwise correlation methods involve the calculation of the correlation coefficient between each pair of nodes (taxonomic entity like Genera) leading to the inclusion of spurious indirect connections. On the other hand, direct association methods use conditional independence to avoid the detection of correlated but indirectly connected taxonomic entities (31, 47). A null model is created by resampling and permuting the counts matrix and recalculating the correlations (see next section for details on network analysis and statistics). These permuted association matrices are used to calculate the significance of the inferred correlations by calculating the *P*-values against this null model (46). Brown’s *P*-value merging method (91) is used for combining *P*-values from the pairwise correlations methods to obtain a consensus *P*-value, which can be used to filter for significance. The permutations and *P*-value calculations are only performed on the correlations-based methods. In the final module of this step, the consensus algorithms are used to create the final consensus network using associations from all the NI methods (except Pearson and Spearman, by default). The outputs of this step are co-occurrence association networks encoded in the JSON format (refer to Supplementary) which can also be exportable to a variety of network formats. The list of tools used in this step, along with their modules and references, is provided in Table 1.

### Consensus network and *P*-value merging

The consensus methods combine networks inferred from both correlation-based and direct association methods. First, for the correlation-based methods, we calculate *P*-values using null models and then merge the *P*-values using Brown’s *P*-value merging method (92, 93). Second, we filter all the inferred networks based on an association strength threshold of 0.1 and a *P*-value cutoff of 0.05. Finally, we apply the consensus algorithms we have developed on these filtered networks. These steps are elaborated on in the subsequent sections.

#### Notation

This section defines the notation used to describe the consensus network algorithm of the MiCoNE pipeline. Note that all networks to be compared were updated to have the same number of nodes.

*w*, is the number of co-occurrence networks to be integrated into the consensus network (by default, it is equal to the total number of NI methods excluding Spearman and Pearson, 8)

*q* is the number of unique nodes across all *w* co-occurrence networks

*N* ^*i*^ is the matrix of edge weights for the *i* ^*th*^ co-occurrence network. This is a *q × q* matrix, where *i ∈* {1, . . . , *w*}. *N* ^*i*^_*a,b*_ represents edge *(a, b)* in network *i*

*P* ^*i*^ is the matrix of *P*-values for all edges of the *i* ^*th*^ co-occurrence network. This is a *q × q* matrix, where *i ∈* {1, . . . , *w*}

${\bar{N}}^{i}$ is the “flattened” version of the adjacency matrix *N* ^*i*^ into a *q^2^ ×* 1 column vector, where all columns are stacked onto each other into a q^2^ long vector. Element ${\bar{N}}_{j}^{i}$ corresponds to the *j* ^*th*^ edge in the *i* ^*th*^ network

${\bar{P}}^{i}$ is the “flattened” version of the adjacency matrix *P* ^*i*^ into a *q^2^ ×* 1 column vector, where all columns are stacked onto each other into a *q* ^*2*^ long vector.

#### Permutations and *P*-value calculation

For all correlation-based methods, k ≤ w, 1000 permutations of the original OTU counts data were generated (46). The correlations in the permuted OTU tables are recalculated using the different correlation-based algorithms. Finally, the *P*-value is determined based on how often a more extreme association is observed for randomly permuted data. Note that all the direct association-based methods used in the study have their own regularization methods built in and, hence, do not need to undergo this procedure.

#### *P*-value merging

The next step in the consensus algorithm workflow is to merge the *P*-values for the networks generated by the correlation-based methods. This step is performed using the Brown’s *P*-value merging method (92, 93).

As described in more detail in the Supplementary and in the original reference (92), the final combined *P*-value is given by


(1) $$\begin{array}{l} {\hat{P}}_{j}=1.0-\Phi_{2f}\left(\psi /c\right)\\ where,\;\;\;\psi =-2\sum_{i=1}^{k}\log({\bar{P}}_{j}^{i})\;\;\;\text{and}\;\;\;\Phi_{2f}=CDF\left(\chi_{2f}^{2}\right) \end{array}$$

where ${\hat{P}}_{j}$ is the combined *P*-value for the edge *j, f* is the number of degrees of freedom, and *c* is a scale factor.

Note that we do not use Pearson and Spearman methods in the *P*-value merging step to determine the consensus network. These methods are only used for demonstration and comparison. The combined *P*-values are used to threshold for significance right before the consensus algorithm is applied to the inferred networks.

#### Consensus methods

The consensus algorithm was designed to increase the precision (number of true positives) at the end of the NI step. For this purpose, we developed two simple algorithms that combine the edges reported by the different NI tools. Both the algorithms make use of a user-defined parameter θ ( 0≤θ≤1 ) in order to threshold the edges from the individual methods. The inputs to both the algorithms are the co-occurrence networks (association matrices) ${\bar{N}}^{i}$ (flattened version of *N* ^*i*^) generated by each method i , and the threshold parameter θ . Here, the ${\bar{N}}^{i}$ each have the same set of nodes *q* and only differ by the value of the association inferred between every pair. Networks that do not have a particular node are updated such that the node is added as an isolated component. In this manner, ${\bar{N}}_{j}^{i}$ represents edge *j* in network *i* .

Note that the consensus method is only used to filter relevant interactions. If a given pair of nodes is inferred to have edges that satisfy the consensus requirements, all corresponding edges from the *w* networks will be returned by the algorithm, as a multigraph. Based on this approach, MiCoNE reports as the default output, the consensus network where each edge is annotated with weights (correlations for the correlation-based methods and direct associations for the other methods) from all the methods used in the consensus algorithm.

Algorithm 1 - **Simple voting (SV):**

The SV method performs a voting-based consensus to determine whether an edge will exist between a given node-pair in the final consensus network (71, 72). For each pair of nodes, we determine the number of NI methods that report an edge *j* between them, i.e., ${\bar{N}}_{j}^{i},\forall i\in \left\{1,\ldots ,w\right\}$ . Each node-pair will have an edge in the final consensus network if the number of reported edges is larger than the threshold (Equation 3).

The number of reported edges is computed as follows:

For each edge *j*, we obtain *M* ^*_j_*^ which represents the number of networks in which edge *j* is reported. Formally, *M* ^*_j_*^ is calculated as the following function:


(2) $$M_{j}=f(g({\bar{N}}_{j}^{i=1}),\ldots ,g({\bar{N}}_{j}^{i=w}))\;$$

where *g* and *f* are defined as follows:


$$g(x)=\left\{ \begin{array}{ll} & 0,\text{ if }x=0,\\ & -1,\text{ if }x<0,\\ & 1,\text{ if }x>0 \end{array}\right.$$

and


$$f\,\left(x_{1},\ldots ,x_{w}\right)=m\,a\,x\,\left(\#\,\left(i|x_{i}=-1\right),\#\,\left(i|x_{i}=1\right)\right)$$

where # refers to the cardinality of the set.

The edge *j* is selected to be present in the final consensus network if the number of networks in which *j* appears is greater than a threshold, i.e.,


(3) $$M_{j}\geq \left\lfloor \theta \times w\right\rfloor$$

where θ is the user-defined threshold parameter.

The SV method returns the union of the networks when $0\leq \theta \leq \frac{1}{w}$ and will return the intersection when $\frac{\left(w-1\right)}{w}\leq \theta \leq 1$ . In general, if $\frac{\left(n-1\right)}{w}\leq \theta \leq \frac{n}{w}$ , this algorithm will report an edge in the consensus network when at least n network inference methods report this edge.

Algorithm 2 - **Scaled-sum (SS) method:**

This algorithm generates a consensus network based on the sum of all edges (weights of associations) reported between a pair of nodes (71, 72). Since in generating a consensus network using this method we sum the edges reported by direct association methods with those from correlation-based methods, summing of the edges is preceded by a pre-processing step, in which all networks are re-scaled.

First, the network generated by each NI method ( ${\bar{N}}^{i}$ ) is re-scaled into a normalized version ( ${\bar{S}}^{i}$ ), as follows:


(4) $${\bar{S}}^{i}=\frac{{\bar{N}}^{i}}{\max\left(|{\bar{N}}^{i}|\right)},\forall i\in 1,\ldots ,w$$

In this way, it is guaranteed that $\max\left(|{\bar{S}}^{i}|\right)=1$ .

Next, for each edge *j* , we sum the weights of all reported edges from the different networks.


(5) $$s_{j}=\sum_{i=1}^{w}{\bar{S}}_{j}^{i}$$

An edge *j* will be included in the consensus network if *s*_*j*_ passes a threshold.


(6) $$|s_{j}|>(w-1)\times \theta$$

The advantage of this method over the SV method is that it also takes into account the strength of the association reported for that particular node in the inferred networks.

### Network variability

#### Notation

This section defines the notation used for the network variability analysis performed for Fig. 6.

*W*, is the number of co-occurrence networks generated from all possible combinations of tools and parameters in the workflow. Note that this is different from *w*, which counted only the different NI modules.

*Q*, is the number of unique nodes across all *W* networks.

*N* ^*i*^ is the edge weights of the *i* ^*th*^ co-occurrence network represented as a *Q × Q* adjacency matrix, where *i ∈* {1, . . . , *W*}. $N_{a,b}^{i}$ represents the edge (*a, b*) in network i

${\bar{N}}^{i}$ is the “flattened” version of the adjacency matrix *N* ^*i*^ into a *Q* ^*2*^ × 1 column vector, where all columns are stacked onto each other into a *Q* ^*2*^ long vector.

### Principal component analysis and variability calculation

In order to compare across different networks and calculate the degree of variability induced by the choice of different modules, we organized multiple networks into a single mathematical structure that we could use for linear regression. First, we obtained the co-occurrence network ${\bar{N}}^{i}$ for each of the *W* possible tool and parameter combinations in the workflow. We then constructed a matrix $\bar{N}$ whose *i*th column is the flattened version of the *i*th network, that is, the column vector ${\bar{N}}^{i}$ . Therefore, ${\bar{N}}_{j}^{i}$ is the weight of edge *j* in the network *i* is assigned a value of 0 if edge *j* did not exist in network *i* but was present in one of the other networks. Note that row *j* of $\bar{N}$ , ${\bar{N}}_{j}$ is the vector that encodes the values of edge *j* across all the networks.


$$\bar{N}=\left[\begin{array}{cccc} {\bar{N}}_{1}^{1} & {\bar{N}}_{1}^{2} & \cdots & {\bar{N}}_{1}^{W}\\ {\bar{N}}_{2}^{1} & {\bar{N}}_{2}^{2} & \cdots & {\bar{N}}_{2}^{W}\\ \vdots & \vdots & \vdots & \vdots \\ {\bar{N}}_{Q^{2}}^{1} & {\bar{N}}_{Q^{2}}^{2} & \cdots & {\bar{N}}_{Q^{2}}^{W} \end{array}\right]$$

To infer the variability contributed due to the different steps in the pipeline, we can perform a linear regression on each edge in $\bar{N}$ and a subsequent ANOVA to extract the within-group variances. A major issue with this approach is that the possibility of correlations existing between the edges of the network could lead to inaccurate estimates of the variance if a linear model were used to directly model the relationships between edges and steps in the workflow. Therefore, in order to remedy this issue, we performed a PCA (principal component analysis) on the matrix $\bar{N}$ to obtain the C matrix ( W×c ) of components for each network, such that we reduce the dimensions from the *Q* ^*2*^ dimensional edge space to a *c* dimensional component space.

We then use linear regression to express each component *C*_*j*_ (where j∈1:c ) as a linear function of categorical variables that describe the possible options in each of the steps of the pipeline.

In particular, we infer parameters $\alpha_{j}$ such that


(7) $$C_{j}=\sum_{i=1}^{5}\left(\alpha_{j}^{DC(i)}\delta_{j}^{DC(i)}\right)+\sum_{i=1}^{2}\left(\alpha_{j}^{CC(i)}\delta_{j}^{CC(i)}\right)+\sum_{i=1}^{3}\left(\alpha_{j}^{TA(i)}\delta_{j}^{TA(i)}\right)\;\begin{array}{l} +\sum_{i=1}^{2}\left(\alpha_{j}^{OP(i)}\delta_{j}^{OP(i)}\right)+\sum_{i=1}^{10}\left(\alpha_{j}^{NI(i)}\delta_{j}^{NI(i)}\right)+\epsilon_{j} \end{array}$$

where $\alpha_{i}$ are the coefficients of the regression, $\epsilon_{i}$ are the residuals, and $\delta_{i}$ are the indicator variables that correspond to the processes utilized in the pipeline used to create the network *N*_*i*_; for example, $\delta_{i}^{D\,C\,\left(1\right)}=1$ if the DC (1) process was used in the generation of the network *N* ^*i*^.

Here,

1. D⁢C⁢(i)∈ {CR, OR, DN, D2, DB}
2. C⁢C⁢(i)∈ {remove bimera, uchime-*de novo*}
3. T⁢A⁢(i)∈ {NaiveBayes(GG), NaiveBayes(SILVA), BLAST(NCBI)}
4. O⁢P⁢(i)∈ {Filter(on), Filter(off)}
5. N⁢I⁢(i)∈ {SparCC, propr, Spearman, Pearson, SpiecEasi, COZINE, HARMONIES, SPRING, mLDM, FlashWeave}

The variance contributed by each step of the pipeline was calculated for every component in C matrix through ANOVA using the Python statsmodels (94) package and is shown in Fig. 6A. The total variance for the network was calculated by adding the variances for each connection and normalizing with the degrees of freedom. The merged network table $\bar{N}$ was used as the input to the PCA to generate Fig. 6B.

### Synthetic interaction data

We generated synthetic interaction data using two methodologies previously used for benchmarking NI methods.

The first method, “seqtime” (73), used generalized Lotka-Volterra (gLV) equations to model the microbial community dynamics and utilized the Klemm-Eguíluz algorithm to generate a clique-based interaction network (26). We used the seqtime R package to simulate communities with number of species (*N*) varying from 10 to 150 (10, 25, 50, 100, 150, and 200). The initial species concentrations were randomly sampled from a Poisson distribution, and the simulation was rerun to generate a number of samples (*S*) varying from 50 to 500 (50, 100, 200, 500) for different communities. The abundance values of the species in the community at the end of the simulation time were used to create the OTU table.

The second method, “NorTA”, used the Normal to Anything (NorTA) approach coupled with a given interaction network topology to generate the abundance distribution of the microbial community (47). We used the spieceasi R package (47) to simulate communities with different network topologies (scale-free, cluster, block, Erdos-Renyi, band, and hub) and target abundance distributions (Negative Binomial, Poisson, Zero-Inflated Negative Binomial). The OTU table was generated using the American Gut Project example in the spieceasi package (amgut1.filt) with the default parameter options.

For each method, we generated the OTU table depicting the abundances of species and used this as input to generate association networks using MiCoNE pipeline. The interaction matrix was used as the source of expected (true) interactions and the associations predicted using MiCoNE were the source of predicted interactions. Finally, for each data set, we evaluated the precision and sensitivity of the associations predicted by the individual NI methods as well as the consensus (Fig. 5; Fig. S5 and S6).

### Statistical analyses

#### DC step

In order to compare the representative sequences generated by the various methods in the DC step, we employed both the weighted (58) (Fig. 2A) and unweighted UniFrac methods (59) (Fig. 2B). The UniFrac distance metric (unique fraction metric) is a beta-diversity measure that computes the distance between two sets of taxa as the fraction of the branch length of the tree that leads to descendants from either one environment or the other, but not both (59). The weighted UniFrac distance metric takes into account the abundances of the representative sequences when calculating shared and unshared branch lengths, whereas the unweighted UniFrac distance metric does not and, hence, gives equal weights to each sequence. In Fig. 2, the distances between methods are the distance between the reference sequence distribution for a pair of methods averaged over every sample in the data set. All UniFrac calculations were performed using the scikit-bio (95) v0.5.6 Python package.

#### TA step

In Fig. 3C, we used the Bray-Curtis distance metric to calculate the distance between the predicted (using the taxonomy databases in the TA step) and expected taxonomic distribution. The Bray-Curtis distance is used to quantify the compositional dissimilarity between two different taxonomic distributions defined by vectors *u* and *v* . It is defined as


$$d=\frac{\sum_{i}|u_{i}-v_{i}|}{\sum_{i}|u_{i}+v_{i}|}$$

The Bray-Curtis distance calculations were performed using the scipy (64) v1.8.0 Python package.

#### NI step

In Fig. 5, we evaluated the precision and sensitivity of the inferred association networks (using the various NI algorithms and the consensus methods) against the original interaction network used to create the taxonomic distribution. We used the following formulations of precision and sensitivity to calculate the accuracy of the predictions:

where TP stands for true positives; FP for false positives. and FN for false negatives.

## Data Availability

Code and data are available as follows: pipeline, https://github.com/segrelab/MiCoNE; documentation, https://micone.readthedocs.io; data and scripts, https://github.com/segrelab/MiCoNE-pipeline-paper; and synthetic data and scripts, https://github.com/segrelab/MiCoNE-synthetic-data.

## SUPPLEMENTAL MATERIAL

Fig. S1 msystems.00961-22-s0001.eps The t-SNE plot of all the inferred networks clusters the networks based on the taxonomy reference database used. Each point on the t-SNE plot represents a network inferred using different combinations of tools and parameters that are available in the MiCoNE pipeline. The points are colored by the tools and parameters used in DC step (A), TA step (B), OP step (C), and NI step (D). The separation of the points based on taxonomy reference database shows that the points cluster based on reference database in high-dimensional space.

Fig. S2 msystems.00961-22-s0002.eps The UniFrac distance between the 1000 most abundant representative sequences is higher than that when all sequences are considered. Each value is the average UniFrac distance between the reference sequences generated by the various methods in the DC step (similar to Fig. 2). There is an increase in both weighted and unweighted UniFrac distances compared to when all the representative sequences are considered. This shows that the 1000 most abundant representative sequences generated by the DC methods are not as similar to each other. And since the weighted UniFrac is much smaller than the unweighted UniFrac distance, we can conclude that those reference sequences that are present in the middle of the abundance distribution (considering all sequences) are dissimilar.

Fig. S3 msystems.00961-22-s0003.eps The weighted and unweighted UniFrac distances are small for the representative sequences generated using remove bimera and uchime for each denoising method. With the exception of *de novo* and open reference under the unweighted UniFrac metric, all the other methods have high similarity, implying that the two CC methods, uchime and remove bimera, produce similar outputs. This is especially true for the DADA2 and Deblur methods which are the recommended denoising methods in the MiCoNE pipeline. Therefore, remove bimera is recommended as the default chimera method if one is using DADA2 and uchime-*de nov*o when one is using Deblur since these methods were developed for these respective algorithms (QIIME2 uses uchime-*de novo* in the Deblur workflow).

Fig. S4 msystems.00961-22-s0004.eps The pairwise comparison of assignments generated using different databases for all representative sequences has a higher proportion of mismatches. The comparison made here is similar to Fig. 3B, but instead of the top 100 taxonomic entities (by abundance), all the assignments from one database are matched with those from the other two databases. The higher percentage of mismatches implies that the assigned taxonomies in the more abundant sequences (top 100) match more consistently.

Fig. S5 msystems.00961-22-s0005.eps The precision and sensitivity of the inferred networks on the “NorTA” synthetic interaction data. The different consensus methods used are SS and SV methods. Pearson and Spearman methods are not used in the calculation of the consensus. Among all the independent NI methods, SpiecEasi has the best average precision (0.944), but the overall best precision was consistently obtained by the SS method (0.956, 0.985, and 1.000). The SV method when using the presence of edges in all inferred networks as a requirement (*θ* = 1.000) also outperforms SpiecEasi on average precision (0.969). Although SpiecEasi has a higher sensitivity, if the goal of NI is to obtain the list of associations that have a high probability of existing in the real microbial community, then the consensus methods perform better.

Fig. S6 msystems.00961-22-s0006.eps The precision and sensitivity of the inferred networks on the “seqtime“ synthetic interaction data. The different consensus methods used are scaled-sum (SS) and simple voting (SV) methods. Pearson and Spearman methods are not used in the calculation of the consensus. Among all the independent network inference methods, SpiecEasi has the best average precision (0.624), but the overall best precision was consistently obtained by the scaled-sum method (0.688, 0.820, and 1.000). The simple voting method when using the presence of edges in all inferred networks as a requirement (*θ* = 1.000) also outperforms SpiecEasi on average precision (0.692). These results show that the SS method is not only much better suited for inferring robust and accurate interactions from count data generated from network topologies (NorTA), but it is also capable of accurately extracting real associations from Lotka-Volterra simulations.

Fig. S7 msystems.00961-22-s0007.eps Sensitivity analysis of the default settings of the MiCoNE pipeline. (A) The network constructed using the default pipeline parameters (DC=DADA2, CC=remove bimera, TA=GG, OP=Filter(on), NI=scaled-sum consensus) is compared against networks generated when one of the steps uses a different tool. The layout is created by fixing the positions of all the nodes from all networks and then drawing only the relevant edges, making the connections directly comparable. The edges colored green are positive associations and those in red are negative associations. We observe that changing the TA and OP steps leads to the creation of the most number of unique edges. (B) The dot plot showing the fraction of nodes (left) and edges (right) in common between the default network and the networks generated by changing one step of the default pipeline. The low value of the common fraction for TA and OP steps shows that these steps induce the biggest changes in nodes and edges. The NI step is not shown in this analysis because the consensus methods use edges from the individual NI methods and a comparison would be biased.

Fig. S8 msystems.00961-22-s0008.eps Network variance analysis of the stool samples from radiation-exposed bank vole (EMP data set). (A) The percentage of variance in the networks contributed by the denoising and clustering (DC), taxonomy assignment (TA), OTU processing (OP), and network inference (NI) steps of the pipeline calculated using ANOVA on a linear model (see Methods). A weight threshold of 0.1 and a *P*-value threshold of 0.05 were applied to each network before the analysis. The taxonomy database contributes most to the variance between the networks (57.96%) followed by the filtering of the counts matrix (33.48%) in the OP step. The variation due to the NI, DC, and CC steps is much smaller in comparison (6.69%, 1.30%, and 0.00%, respectively). The negligible fraction labeled as the residual is an artifact that arises when multiple steps are changed at the same time. (B) All the inferred networks generated from various combinations of tools are shown as points on a PCA plot. Each point on the PCA plot represents a network inferred using different combinations of tools and parameters that are available in the MiCoNE pipeline. The color of the points corresponds to the tools used at each step of the pipeline (DC, TA, OP, and NI). The points on the PCA plot can be largely grouped based on the TA step, and the extent of this separation decreases when the filtering is turned on in the OP step. However, unlike Fig. 6B, here, we observe that the networks that utilize GG and NCBI databases have the variation along similar axes in the two-dimensional PCA plot. This could imply that the taxonomy assignments returned by these two databases for this data set might be more similar to each other compared to those in the FMT data set.

Table S1 msystems.00961-22-s0009.pdf Table of global network metrics for networks inferred from all possible combinations of tools. In each row, one tool in a particular step is kept constant, and the metric is calculated for every possible combination of tools for the other steps of the pipeline. Therefore, each row shows the grouped average metric for each tool in every step of the pipeline. The network inference methods show the most variation in the global network metrics compared to tools in other steps of the pipeline.

Supplemental Text msystems.00961-22-s0010.pdf Supplemental text describing data processing, synthetic interaction data generation, network metrics, *P*-value merging, additional network variance analysis, and supplemental discussion sections.

ASM does not own the copyrights to Supplemental Material that may be linked to, or accessed through, an article. The authors have granted ASM a non-exclusive, world-wide license to publish the Supplemental Material files. Please contact the corresponding author directly for reuse.

## References

[1] Lima-Mendez G , Faust K , Henry N , Decelle J , Colin S , Carcillo F , Chaffron S , Ignacio-Espinosa JC , Roux S , Vincent F , Bittner L , Darzi Y , Wang J , Audic S , Berline L , Bontempi G , Cabello AM , Coppola L , Cornejo-Castillo FM , d’Ovidio F , De Meester L , Ferrera I , Garet-Delmas M-J , Guidi L , Lara E , Pesant S , Royo-Llonch M , Salazar G , Sánchez P , Sebastian M , Souffreau C , Dimier C , Picheral M , Searson S , Kandels-Lewis S , Tara Oceans coordinators, Gorsky G , Not F , Ogata H , Speich S , Stemmann L , Weissenbach J , Wincker P , Acinas SG , Sunagawa S , Bork P , Sullivan MB , Karsenti E , Bowler C , de Vargas C , Raes J . 2015. Ocean Plankton. determinants of community structure in the global Plankton Interactome. Science 348:1262073. doi:10.1126/science.1262073 25999517

[2] Thompson LR , Sanders JG , McDonald D , Amir A , Ladau J , Locey KJ , Prill RJ , Tripathi A , Gibbons SM , Ackermann G , Navas-Molina JA , Janssen S , Kopylova E , Vázquez-Baeza Y , González A , Morton JT , Mirarab S , Zech Xu Z , Jiang L , Haroon MF , Kanbar J , Zhu Q , Jin Song S , Kosciolek T , Bokulich NA , Lefler J , Brislawn CJ , Humphrey G , Owens SM , Hampton-Marcell J , Berg-Lyons D , McKenzie V , Fierer N , Fuhrman JA , Clauset A , Stevens RL , Shade A , Pollard KS , Goodwin KD , Jansson JK , Gilbert JA , Knight R , Earth Microbiome Project Consortium . 2017. A communal catalogue reveals earth’s Multiscale microbial Diver- Sity. Nature 551:457–463. doi:10.1038/nature24621 29088705 PMC6192678

[3] Royo-Llonch M , Sánchez P , Ruiz-González C , Salazar G , Pedrós-Alió C , Sebastián M , Labadie K , Paoli L , M Ibarbalz F , Zinger L , Churcheward B , Tara Oceans Coordinators, Chaffron S , Eveillard D , Karsenti E , Sunagawa S , Wincker P , Karp-Boss L , Bowler C , Acinas SG . 2021. Compendium of 530 metagenome-assembled bacterial and archaeal genomes from the polar Arctic ocean. Nat Microbiol 6:1561–1574. doi:10.1038/s41564-021-00979-9 34782724

[4] Tedersoo L , Bahram M , Põlme S , Kõljalg U , Yorou NS , Wijesundera R , Villarreal Ruiz L , Vasco-Palacios AM , Thu PQ , Suija A , Smith ME , Sharp C , Saluveer E , Saitta A , Rosas M , Riit T , Ratkowsky D , Pritsch K , Põldmaa K , Piepenbring M , Phosri C , Peterson M , Parts K , Pärtel K , Otsing E , Nouhra E , Njouonkou AL , Nilsson RH , Morgado LN , Mayor J , May TW , Majuakim L , Lodge DJ , Lee SS , Larsson K-H , Kohout P , Hosaka K , Hiiesalu I , Henkel TW , Harend H , Guo L , Greslebin A , Grelet G , Geml J , Gates G , Dunstan W , Dunk C , Drenkhan R , Dearnaley J , De Kesel A , Dang T , Chen X , Buegger F , Brearley FQ , Bonito G , Anslan S , Abell S , Abarenkov K . 2014. Fungal biogeography. Global diversity and geography of soil fungi. Science 346:1256688. doi:10.1126/science.1256688 25430773

[5] Danko D , Bezdan D , Afshin EE , Ahsanuddin S , Bhattacharya C , Butler DJ , Chng KR , Donnellan D , Hecht J , Jackson K , Kuchin K , Karasikov M , Lyons A , Mak L , Meleshko D , Mustafa H , Mutai B , Neches RY , Ng A , Nikolayeva O , Nikolayeva T , Png E , Ryon KA , Sanchez JL , Shaaban H , Sierra MA , Thomas D , Young B , Abudayyeh OO , Alicea J , Bhattacharyya M , Blekhman R , Castro-Nallar E , Cañas AM , Chatziefthimiou AD , Crawford RW , De Filippis F , Deng Y , Desnues C , Dias-Neto E , Dybwad M , Elhaik E , Ercolini D , Frolova A , Gankin D , Gootenberg JS , Graf AB , Green DC , Hajirasouliha I , Hastings JJA , Hernandez M , Iraola G , Jang S , Kahles A , Kelly FJ , Knights K , Kyrpides NC , Łabaj PP , Lee PKH , Leung MHY , Ljungdahl PO , Mason-Buck G , McGrath K , Meydan C , Mongodin EF , Moraes MO , Nagarajan N , Nieto-Caballero M , Noushmehr H , Oliveira M , Ossowski S , Osuolale OO , Özcan O , Paez-Espino D , Rascovan N , Richard H , Rätsch G , Schriml LM , Semmler T , Sezerman OU , Shi L , Shi T , Siam R , Song LH , Suzuki H , Court DS , Tighe SW , Tong X , Udekwu KI , Ugalde JA , Valentine B , Vassilev DI , Vayndorf EM , Velavan TP , Wu J , Zambrano MM , Zhu J , Zhu S , Mason CE , International MetaSUB Consortium . 2021. A global metagenomic map of urban microbiomes and antimicrobial resistance. Cell 184:3376–3393. doi:10.1016/j.cell.2021.05.002 34043940 PMC8238498

[6] McLellan SL , Fisher JC , Newton RJ . 2015. The microbiome of urban waters. Int Microbiol 18:141–149. doi:10.2436/20.1501.01.244 27036741 PMC8793681

[7] Human Microbiome Project Consortium . 2012. A framework for human microbiome research. Nature 486:215–221. doi:10.1038/nature11209 22699610 PMC3377744

[8] Zelezniak A , Andrejev S , Ponomarova O , Mende DR , Bork P , Patil KR . 2015. Metabolic dependencies drive species Co-occurrence in diverse microbial communities. Proc Natl Acad Sci U S A 112:6449–6454. doi:10.1073/pnas.1421834112 25941371 PMC4443341

[9] Ghoul M , Mitri S . 2016. The ecology and evolution of microbial competition. Trends Microbiol 24:833–845. doi:10.1016/j.tim.2016.06.011 27546832

[10] Coyte KZ , Rakoff-Nahoum S . 2019. Understanding competition and cooperation within the mammalian gut microbiome. Curr Biol 29:R538–R544. doi:10.1016/j.cub.2019.04.017 31163167 PMC6935513

[11] D’Souza G , Shitut S , Preussger D , Yousif G , Waschina S , Kost C . 2018. Ecology and evolution of metabolic cross-feeding interactions in bacteria. Nat Prod Rep 35:455–488. doi:10.1039/c8np00009c 29799048

[12] Hu T , Chitnis N , Monos D , Dinh A . 2021. Next-generation sequencing technologies: an overview. Hum Immunol 82:801–811. doi:10.1016/j.humimm.2021.02.012 33745759

[13] Buermans HPJ , den Dunnen JT . 2014. Next generation sequencing technology: advances and applications. Biochim Biophys Acta 1842:1932–1941. doi:10.1016/j.bbadis.2014.06.015 24995601

[14] Narihiro T , Kamagata Y . 2017. Genomics and metagenomics in microbial ecology: recent advances and challenges. Microbes Environ 32:1–4. doi:10.1264/jsme2.ME3201rh 28367917 PMC5371069

[15] Ju F , Zhang T . 2015. 16S rRNA gene high-throughput sequencing data mining of microbial diversity and interactions. Appl Microbiol Biotechnol 99:4119–4129. doi:10.1007/s00253-015-6536-y 25808518

[16] Jovel J , Patterson J , Wang W , Hotte N , O’Keefe S , Mitchel T , Perry T , Kao D , Mason AL , Madsen KL , Wong G-S . 2016. Characterization of the gut microbiome using 16S or shotgun metagenomics. Front Microbiol 7:459. doi:10.3389/fmicb.2016.00459 27148170 PMC4837688

[17] Quince C , Walker AW , Simpson JT , Loman NJ , Segata N . 2017. Corrigendum: shotgun metagenomics, from sampling to analysis. Nat Biotechnol 35:1211. doi:10.1038/nbt1217-1211b 29220029

[18] Sedlar K , Kupkova K , Provaznik I . 2017. Bioinformatics strategies for taxonomy independent binning and visualization of sequences in shotgun metagenomics. Comput Struct Biotechnol J 15:48–55. doi:10.1016/j.csbj.2016.11.005 27980708 PMC5148923

[19] Gilbert JA , Meyer F , Antonopoulos D , Balaji P , Brown CT , Brown CT , Desai N , Eisen JA , Evers D , Field D , Feng W , Huson D , Jansson J , Knight R , Knight J , Kolker E , Konstantindis K , Kostka J , Kyrpides N , Mackelprang R , McHardy A , Quince C , Raes J , Sczyrba A , Shade A , Stevens R . 2010. Meeting report: the terabase metagenomics workshop and the vision of an earth microbiome project. Stand Genomic Sci 3:243–248. doi:10.4056/sigs.1433550 21304727 PMC3035311

[20] Proctor LM . 2019. The integrative human microbiome project. Nature 569:641–648. doi:10.1038/s41586-019-1238-8 31142853 PMC6784865

[21] Lloyd-Price J , Abu-Ali G , Huttenhower C . 2016. The healthy human microbiome. Genome Med 8:51. doi:10.1186/s13073-016-0307-y 27122046 PMC4848870

[22] Lubbe A , Bowen BP , Northen T . 2017. Exometabolomic analysis of cross-feeding metabolites. Metabolites 7:50. doi:10.3390/metabo7040050 28976938 PMC5746730

[23] Jian X , Guo X , Wang J , Tan ZL , Xing X-H , Wang L , Zhang C . 2020. Microbial Microdroplet Culture system (MMC): an integrated platform for automated, high-throughput microbial cultivation and adaptive evolution. Biotechnol Bioeng 117:1724–1737. doi:10.1002/bit.27327 32159223

[24] Hsu RH , Clark RL , Tan JW , Ahn JC , Gupta S , Romero PA , Venturelli OS . 2019. Microbial interaction network inference in microﬂuidic droplets. Cell Syst 9:229–242. doi:10.1016/j.cels.2019.06.008 31494089 PMC6763379

[25] Zuñiga C , Zaramela L , Zengler K . 2017. Elucidation of complexity and prediction of interactions in microbial communities. Microb Biotechnol 10:1500–1522. doi:10.1111/1751-7915.12855 28925555 PMC5658597

[26] Röttjers L , Faust K . 2018. From hairballs to hypotheses–biological insights from microbial networks. FEMS Microbiol Rev 42:761–780. doi:10.1093/femsre/fuy030 30085090 PMC6199531

[27] Faust K , Sathirapongsasuti JF , Izard J , Segata N , Gevers D , Raes J , Huttenhower C . 2012. Microbial co-occurrence relationships in the human Microbiome. PLoS Comput Biol 8:e1002606. doi:10.1371/journal.pcbi.1002606 22807668 PMC3395616

[28] Lee KK , Kim H , Lee Y-H . 2022. Cross-Kingdom co-occurrence networks in the plant microbiome: Importance and ecological interpretations. Front Microbiol 13:953300. doi:10.3389/fmicb.2022.953300 35958158 PMC9358436

[29] Faust K , Raes J . 2012. Microbial interactions: From networks to models. Nat Rev Microbiol 10:538–550. doi:10.1038/nrmicro2832 22796884

[30] Ma B , Wang Y , Ye S , Liu S , Stirling E , Gilbert JA , Faust K , Knight R , Jansson JK , Cardona C , Röttjers L , Xu J . 2020. Earth microbial co-occurrence network reveals interconnection pattern across microbiomes. Microbiome 8:82. doi:10.1186/s40168-020-00857-2 32498714 PMC7273686

[31] Menon R , Ramanan V , Korolev KS . 2018. Interactions between species introduce spurious associations in microbiome studies. PLoS Comput Biol 14:e1005939. doi:10.1371/journal.pcbi.1005939 29338008 PMC5786326

[32] Gilbert JA , Quinn RA , Debelius J , Xu ZZ , Morton J , Garg N , Jansson JK , Dorrestein PC , Knight R . 2016. Microbiome-wide association studies link dynamic microbial consortia to disease. Nature 535:94–103. doi:10.1038/nature18850 27383984

[33] Wu GD , Compher C , Chen EZ , Smith SA , Shah RD , Bittinger K , Chehoud C , Albenberg LG , Nessel L , Gilroy E , Star J , Weljie AM , Flint HJ , Metz DC , Bennett MJ , Li H , Bushman FD , Lewis JD . 2016. Comparative metabolomics in vegans and omnivores reveal constraints on diet-dependent gut microbiota metabolite production. Gut 65:63–72. doi:10.1136/gutjnl-2014-308209 25431456 PMC4583329

[34] Wang B , Yao M , Lv L , Ling Z , Li L . 2017. The human microbiota in health and disease. Engineering 3:71–82. doi:10.1016/J.ENG.2017.01.008

[35] Belizário JE , Napolitano M . 2015. Human microbiomes and their roles in dysbiosis, common diseases, and novel therapeutic approaches. Front Microbiol 6:1050. doi:10.3389/fmicb.2015.01050 26500616 PMC4594012

[36] Fierer N . 2017. Embracing the unknown: disentangling the complexities of the soil microbiome. Nat Rev Microbiol 15:579–590. doi:10.1038/nrmicro.2017.87 28824177

[37] Jiao S , Chen W , Wei G . 2019. Resilience and assemblage of soil microbiome in response to chemical contamination combined with plant growth. Appl Environ Microbiol 85:e02523-18. doi:10.1128/AEM.02523-18 30658982 PMC6414375

[38] Friedman J , Alm EJ . 2012. Inferring correlation networks from genomic survey data. PLoS Comput Biol 8:e1002687. doi:10.1371/journal.pcbi.1002687 23028285 PMC3447976

[39] Bharti R , Grimm DG . 2019. Current challenges and best-practice protocols for microbiome analysis. Brief Bioinform 22:1–6. doi:10.1093/bib/bbz155 PMC7820839 31848574

[40] Pollock J , Glendinning L , Wisedchanwet T , Watson M . 2018. The madness of microbiome: attempting to find consensus "best practice" for 16S microbiome studies. Appl Environ Microbiol 84:e02627-17. doi:10.1128/AEM.02627-17 29427429 PMC5861821

[41] Caporaso JG , Kuczynski J , Stombaugh J , Bittinger K , Bushman FD , Costello EK , Fierer N , Peña AG , Goodrich JK , Gordon JI , Huttley GA , Kelley ST , Knights D , Koenig JE , Ley RE , Lozupone CA , McDonald D , Muegge BD , Pirrung M , Reeder J , Sevinsky JR , Turnbaugh PJ , Walters WA , Widmann J , Yatsunenko T , Zaneveld J , Knight R . 2010. QIIME allows analysis of high-throughput community sequencing data. Nat Methods 7:335–336. doi:10.1038/nmeth.f.303 20383131 PMC3156573

[42] Callahan BJ , McMurdie PJ , Rosen MJ , Han AW , Johnson AJA , Holmes SP . 2016. Dada2: high-resolution sample inference from Illumina Amplicon data. Nat Methods 13:581–583. doi:10.1038/nmeth.3869 27214047 PMC4927377

[43] Amir A , McDonald D , Navas-Molina JA , Kopylova E , Morton JT , Zech Xu Z , Kightley EP , Thompson LR , Hyde ER , Gonzalez A , Knight R . 2017. Deblur rapidly resolves single-nucleotide community sequence patterns. mSystems 2:e00191-16. doi:10.1128/mSystems.00191-16 28289731 PMC5340863

[44] Bokulich NA , Kaehler BD , Rideout JR , Dillon M , Bolyen E , Knight R , Huttley GA , Gregory Caporaso J . 2018. Optimizing taxonomic classification of marker-gene Amplicon sequences with QIIME 2's Q2-feature-Classifier Plugin. Microbiome 6:90. doi:10.1186/s40168-018-0470-z 29773078 PMC5956843

[45] Weiss S , Xu ZZ , Peddada S , Amir A , Bittinger K , Gonzalez A , Lozupone C , Zaneveld JR , Vázquez-Baeza Y , Birmingham A , Hyde ER , Knight R . 2017. Normalization and microbial differential abundance strategies depend upon data characteristics. Microbiome 5:27. doi:10.1186/s40168-017-0237-y 28253908 PMC5335496

[46] Watts SC , Ritchie SC , Inouye M , Holt KE . 2019. Fastspar: rapid and Scalable correlation estimation for compositional data. Bioinformatics 35:1064–1066. doi:10.1093/bioinformatics/bty734 30169561 PMC6419895

[47] Kurtz ZD , Müller CL , Miraldi ER , Littman DR , Blaser MJ , Bonneau RA . 2015. Sparse and compositionally robust inference of microbial ecological networks. PLoS Comput Biol 11:e1004226. doi:10.1371/journal.pcbi.1004226 25950956 PMC4423992

[48] Tackmann J , Matias Rodrigues JF , von Mering C . 2019. Rapid inference of direct interactions in large-scale ecological networks from heterogeneous microbial sequencing data. Cell Syst 9:286–296. doi:10.1016/j.cels.2019.08.002 31542415

[49] Keegan KP , Glass EM , Meyer F . 2016. MG-RAST, a metagenomics service for analysis of microbial community structure and function. Methods Mol Biol 1399:207–233. doi:10.1007/978-1-4939-3369-3_13 26791506

[50] Gonzalez A , Navas-Molina JA , Kosciolek T , McDonald D , Vázquez-Baeza Y , Ackermann G , DeReus J , Janssen S , Swafford AD , Orchanian SB , Sanders JG , Shorenstein J , Holste H , Petrus S , Robbins-Pianka A , Brislawn CJ , Wang M , Rideout JR , Bolyen E , Dillon M , Caporaso JG , Dorrestein PC , Knight R . 2018. Qiita: rapid, web-enabled Microbiome meta-analysis. Nat Methods 15:796–798. doi:10.1038/s41592-018-0141-9 30275573 PMC6235622

[51] Bolyen E , Rideout JR , Dillon MR , Bokulich NA , Abnet CC , Al-Ghalith GA , Alexander H , Alm EJ , Arumugam M , Asnicar F , Bai Y , Bisanz JE , Bittinger K , Brejnrod A , Brislawn CJ , Brown CT , Callahan BJ , Caraballo-Rodríguez AM , Chase J , Cope EK , Da Silva R , Diener C , Dorrestein PC , Douglas GM , Durall DM , Duvallet C , Edwardson CF , Ernst M , Estaki M , Fouquier J , Gauglitz JM , Gibbons SM , Gibson DL , Gonzalez A , Gorlick K , Guo J , Hillmann B , Holmes S , Holste H , Huttenhower C , Huttley GA , Janssen S , Jarmusch AK , Jiang L , Kaehler BD , Kang KB , Keefe CR , Keim P , Kelley ST , Knights D , Koester I , Kosciolek T , Kreps J , Langille MGI , Lee J , Ley R , Liu Y-X , Loftfield E , Lozupone C , Maher M , Marotz C , Martin BD , McDonald D , McIver LJ , Melnik AV , Metcalf JL , Morgan SC , Morton JT , Naimey AT , Navas-Molina JA , Nothias LF , Orchanian SB , Pearson T , Peoples SL , Petras D , Preuss ML , Pruesse E , Rasmussen LB , Rivers A , Robeson MS , Rosenthal P , Segata N , Shaffer M , Shiffer A , Sinha R , Song SJ , Spear JR , Swafford AD , Thompson LR , Torres PJ , Trinh P , Tripathi A , Turnbaugh PJ , Ul-Hasan S , van der Hooft JJJ , Vargas F , Vázquez-Baeza Y , Vogtmann E , von Hippel M , Walters W , Wan Y , Wang M , Warren J , Weber KC , Williamson CHD , Willis AD , Xu ZZ , Zaneveld JR , Zhang Y , Zhu Q , Knight R , Caporaso JG . 2019. Author correction: reproducible, interactive, scalable and extensible microbiome data science using QIIME 2. Nat Biotechnol 37:1091. doi:10.1038/s41587-019-0252-6 PMC7015180 31341288

[52] Golob JL , Margolis E , Hoffman NG , Fredricks DN . 2017. Evaluating the accuracy of amplicon-based microbiome computational pipelines on simulated human gut microbial communities. BMC Bioinformatics 18:283. doi:10.1186/s12859-017-1690-0 28558684 PMC5450146

[53] Weiss S , Van Treuren W , Lozupone C , Faust K , Friedman J , Deng Y , Xia LC , Xu ZZ , Ursell L , Alm EJ , Birmingham A , Cram JA , Fuhrman JA , Raes J , Sun F , Zhou J , Knight R . 2016. Correlation detection strategies in microbial data SETS vary widely in sensitivity and precision. ISME J 10:1669–1681. doi:10.1038/ismej.2015.235 26905627 PMC4918442

[54] Hu Z , Kishore D , Wang Y , Birzu G , DeLisi C , Korolev K , Segrè D . 2017. A resource for the comparison and integration of heterogeneous microbiome networks. Systematic biology. doi:10.1101/2022.08.07.503059

[55] Kang D-W , Adams JB , Gregory AC , Borody T , Chittick L , Fasano A , Khoruts A , Geis E , Maldonado J , McDonough-Means S , Pollard EL , Roux S , Sadowsky MJ , Lipson KS , Sullivan MB , Caporaso JG , Krajmalnik-Brown R . 2017. Microbiota transfer therapy alters gut ecosystem and improves gastrointestinal and autism symptoms: an open-label study. Microbiome 5:10. doi:10.1186/s40168-016-0225-7 28122648 PMC5264285

[56] Bokulich NA , Rideout JR , Mercurio WG , Shiffer A , Wolfe B , Maurice CF , Dutton RJ , Turnbaugh PJ , Knight R , Caporaso JG . 2016. Mockrobiota: a public resource for microbiome bioinformatics benchmarking. mSystems 1:e00062-16. doi:10.1128/mSystems.00062-16 27822553 PMC5080401

[57] DeSantis TZ , Hugenholtz P , Larsen N , Rojas M , Brodie EL , Keller K , Huber T , Dalevi D , Hu P , Andersen GL . 2006. Greengenes, a chimera-checked 16S rRNA gene database and workbench compatible with ARB. Appl Environ Microbiol 72:5069–5072. doi:10.1128/AEM.03006-05 16820507 PMC1489311

[58] Lozupone CA , Hamady M , Kelley ST , Knight R . 2007. Quantitative and qualitative beta diversity measures lead to diﬀerent insights into factors that structure microbial communities. Appl Environ Microbiol 73:1576–1585. doi:10.1128/AEM.01996-06 17220268 PMC1828774

[59] Lozupone C , Knight R . 2005. Unifrac: a new Phylogenetic method for comparing microbial communities. Appl Environ Microbiol 71:8228–8235. doi:10.1128/AEM.71.12.8228-8235.2005 16332807 PMC1317376

[60] Nearing JT , Douglas GM , Comeau AM , Langille MGI . 2018. Denoising the Denoisers: an independent evaluation of microbiome sequence error-correction approaches. PeerJ 6:e5364. doi:10.7717/peerj.5364 30123705 PMC6087418

[61] Quast C , Pruesse E , Yilmaz P , Gerken J , Schweer T , Yarza P , Peplies J , Glöckner FO . 2013. The SILVA ribosomal RNA Gene database project: improved data processing and web-based tools. Nucleic Acids Res 41:D590–D596. doi:10.1093/nar/gks1219 23193283 PMC3531112

[62] Sayers EW , Barrett T , Benson DA , Bryant SH , Canese K , Chetvernin V , Church DM , DiCuccio M , Edgar R , Federhen S , Feolo M , Geer LY , Helmberg W , Kapustin Y , Landsman D , Lipman DJ , Madden TL , Maglott DR , Miller V , Mizrachi I , Ostell J , Pruitt KD , Schuler GD , Sequeira E , Sherry ST , Shumway M , Sirotkin K , Souvorov A , Starchenko G , Tatusova TA , Wagner L , Yaschenko E , Ye J . 2009. Database resources of the National center for biotechnology information. Nucleic Acids Res 37:D5–D15. doi:10.1093/nar/gkn741 18940862 PMC2686545

[63] Balvočiūtė M , Huson DH . 2017. SILVA, RDP, Greengenes, NCBI and OTT - how do these taxonomies compare? BMC Genomics 18:114. doi:10.1186/s12864-017-3501-4 28361695 PMC5374703

[64] Virtanen P , Gommers R , Oliphant TE , Haberland M , Reddy T , Cournapeau D , Burovski E , Peterson P , Weckesser W , Bright J , van der Walt SJ , Brett M , Wilson J , Millman KJ , Mayorov N , Nelson ARJ , Jones E , Kern R , Larson E , Carey CJ , Polat İ , Feng Y , Moore EW , VanderPlas J , Laxalde D , Perktold J , Cimrman R , Henriksen I , Quintero EA , Harris CR , Archibald AM , Ribeiro AH , Pedregosa F , van Mulbregt P , SciPy 1.0 Contributors . 2020. Scipy 1.0: fundamental Algorithms for scientiﬁc computing in python. Nat Methods 17:261–272. doi:10.1038/s41592-020-0772-5 32015543 PMC7056644

[65] Quinn TP , Richardson MF , Lovell D , Crowley TM . 2017. Propr: an R-package for identifying proportionally abundant features using compositional data analysis. Sci Rep 7:16252. doi:10.1038/s41598-017-16520-0 29176663 PMC5701231

[66] Ha MJ , Kim J , Galloway-Peña J , Do K-A , Peterson CB . 2020. Compositional zero-inflated network estimation for microbiome data. BMC Bioinformatics 21:581. doi:10.1186/s12859-020-03911-w 33371887 PMC7768662

[67] Jiang S , Xiao G , Koh AY , Chen Y , Yao B , Li Q , Zhan X . 2020. HARMONIES: a hybrid approach for Microbiome networks inference via exploiting Sparsity. Front Genet 11:445. doi:10.3389/fgene.2020.00445 32582274 PMC7283552

[68] Yoon G , Gaynanova I , Müller CL . 2019. Microbial networks in SPRING - semi-parametric rank-based correlation and partial correlation estimation for quantitative microbiome data. Front Genet 10:516. doi:10.3389/fgene.2019.00516 31244881 PMC6563871

[69] Yang Y , Chen N , Chen T . 2017. Inference of environmental factor-microbe and microbe- microbe associations from metagenomic data using a hierarchical bayesian statistical model. Cell Syst 4:129–137. doi:10.1016/j.cels.2016.12.012 28125788

[70] Lex A , Gehlenborg N , Strobelt H , Vuillemot R , Pfister H . 2014. Upset: visualization of intersecting SETS. IEEE Trans Vis Comput Graph 20:1983–1992. doi:10.1109/TVCG.2014.2346248 26356912 PMC4720993

[71] Bustince H , Herrera F , Montero J , eds. 2008. Fuzzy sets and their extensions: Representation, aggregation, and models: intelligent systems from decision making to data mining, web intelligence, and computer vision, . In Studies in Fuzziness and soft computing. Vol. 220. Springer. doi:10.1007/978-3-540-73723-0

[72] Tsarev RY , Durmuş MS , Üstoglu I , Morozov VA . 2018. Application of majority voting and consensus voting Algorithms in N-version software. J Phys Conf Ser 1015:042059. doi:10.1088/1742-6596/1015/4/042059

[73] Faust K , Bauchinger F , Laroche B , de Buyl S , Lahti L , Washburne AD , Gonze D , Widder S . 2018. Signatures of ecological processes in microbial community time series. Microbiome 6:120. doi:10.1186/s40168-018-0496-2 29954432 PMC6022718

[74] Shaffer JP , Nothias L-F , Thompson LR , Sanders JG , Salido RA , Couvillion SP , Brejnrod AD , Lejzerowicz F , Haiminen N , Huang S , Lutz HL , Zhu Q , Martino C , Morton JT , Karthikeyan S , Nothias-Esposito M , Dührkop K , Böcker S , Kim HW , Aksenov AA , Bittremieux W , Minich JJ , Marotz C , Bryant MM , Sanders K , Schwartz T , Humphrey G , Vásquez-Baeza Y , Tripathi A , Parida L , Carrieri AP , Beck KL , Das P , González A , McDonald D , Ladau J , Karst SM , Albertsen M , Ackermann G , DeReus J , Thomas T , Petras D , Shade A , Stegen J , Song SJ , Metz TO , Swafford AD , Dorrestein PC , Jansson JK , Gilbert JA , Knight R , Earth Microbiome Project 500 (EMP500) Consortium . 2022. Standardized multi-omics of earth’s Microbiomes reveals microbial and metabolite diversity. Nat Microbiol 7:2128–2150. doi:10.1038/s41564-022-01266-x 36443458 PMC9712116

[75] Ho LKH , Tong VJW , Syn N , Nagarajan N , Tham EH , Tay SK , Shorey S , Tambyah PA , Law ECN . 2020. Gut microbiota changes in children with autism spectrum disorder: a systematic review. Gut Pathog 12:6. doi:10.1186/s13099-020-0346-1 32025243 PMC6996179

[76] Chi L , Cheng X , Lin L , Yang T , Sun J , Feng Y , Liang F , Pei Z , Teng W . 2021. Porphyromonas Gingivalis-Induced cognitive impairment is associated with gut dysbiosis, neuroinflammation, and glymphatic dysfunction. Front Cell Infect Microbiol 11:755925. doi:10.3389/fcimb.2021.755925 34926316 PMC8672439

[77] Shannon P , Markiel A , Ozier O , Baliga NS , Wang JT , Ramage D , Amin N , Schwikowski B , Ideker T . 2003. Cytoscape: A software environment for integrated models of biomolecular interaction networks. Genome Res 13:2498–2504. doi:10.1101/gr.1239303 14597658 PMC403769

[78] Himsolt M . 2010. GML: A portable graph file format. https://tinyurl.com/GMLpdf.

[79] Peschel S , Müller CL , von Mutius E , Boulesteix A-L , Depner M . 2020. NetCoMi: network construction and comparison for microbiome data in R. Bioinformatics. doi:10.1101/2020.07.15.195248 PMC8293835 33264391

[80] Fisher CK , Mehta P . 2014. Identifying keystone species in the human gut microbiome from metagenomic timeseries using sparse linear regression. PLoS One 9:e102451. doi:10.1371/journal.pone.0102451 25054627 PMC4108331

[81] Pacheco AR , Pauvert C , Kishore D , Segrè D . 2022. Toward FAIR representations of microbial interactions. mSystems 7:e0065922. doi:10.1128/msystems.00659-22 36005399 PMC9599284

[82] Hirano H , Takemoto K . 2019. Difficulty in Inferring microbial community structure based on co-occurrence network approaches. BMC Bioinformatics 20:329. doi:10.1186/s12859-019-2915-1 31195956 PMC6567618

[83] Goberna M , Verdú M . 2022. Cautionary notes on the use of co-occurrence networks in soil ecology. Soil Biol Biochem 166:108534. doi:10.1016/j.soilbio.2021.108534

[84] Sung J , Kim S , Cabatbat JJT , Jang S , Jin Y-S , Jung GY , Chia N , Kim P-J . 2017. Global metabolic interaction network of the human gut microbiota for context-specific community-scale analysis. Nat Commun 8:15393. doi:10.1038/ncomms15393 28585563 PMC5467172

[85] Qiita - open-source microbial study management platform. 2015 https://qiita.ucsd.edu/.

[86] Di Tommaso P , Chatzou M , Floden EW , Barja PP , Palumbo E , Notredame C , Floden E . 2017. Nextflow enables reproducible computational Workflows. Nat Biotechnol 35:316–319. doi:10.1038/nbt.3820 28398311

[87] Robeson MS , O’Rourke DR , Kaehler BD , Ziemski M , Dillon MR , Foster JT , Bokulich NA , Pertea M . 2021. Rescript: reproducible sequence taxonomy reference database man- agement. PLOS Comput Biol 17:e1009581. doi:10.1371/journal.pcbi.1009581 34748542 PMC8601625

[88] McMurdie PJ , Holmes S , McHardy AC . 2014. Want not: why rarefying microbiome data is inadmissible. PLOS Comput Biol 10:e1003531. doi:10.1371/journal.pcbi.1003531 24699258 PMC3974642

[89] Chao A , Jost L . 2012. Coverage-based Rarefaction and extrapolation: Standardizing samples by completeness rather than size. Ecology 93:2533–2547. doi:10.1890/11-1952.1 23431585

[90] McDonald D , Clemente JC , Kuczynski J , Rideout JR , Stombaugh J , Wendel D , Wilke A , Huse S , Hufnagle J , Meyer F , Knight R , Caporaso JG . 2012. The biological observation matrix (BIOM) format or: how I learned to stop worrying and love the OME-OME. Gigascience 1:7. doi:10.1186/2047-217X-1-7 23587224 PMC3626512

[91] Brown MB . 1975. 400: A method for combining non-independent, one-sided tests of Sig- Niﬁcance. Biometrics 31:987. doi:10.2307/2529826

[92] Poole W , Gibbs DL , Shmulevich I , Bernard B , Knijnenburg TA . 2016. Combining dependent P-values with an empirical adaptation of brown's method. Bioinformatics 32:i430–i436. doi:10.1093/bioinformatics/btw438 27587659 PMC5013915

[93] Faust K , Raes J . 2016. Conet App: inference of biological association networks using cytoscape. F1000Res 5:1519. doi:10.12688/f1000research.9050.2 27853510 PMC5089131

[94] Seabold S , Perktold J . 2010. Statsmodels: econometric and statistical modeling with python Python in Science Conference; Austin, Texas: . doi:10.25080/Majora-92bf1922-011

[95] Scikit-bio development team, T . 2022. Scikit-bio: A Bioinformatics library for data scientists, students, and developers version 0.5.7

