# Supplementary material for: Inferring microbial co-occurrence networks from amplicon data: a systematic evaluation
Source: mSystems. 2023 Jun 20;8(4):e00961-22. doi: 10.1128/msystems.00961-22 (PMC10469762; doi:10.1128/msystems.00961-22)
Supplement: Table S1 — Table of global network metrics for networks inferred from all possible combinations of tools. In each row, one tool in a particular step is kept constant, and the metric is calculated for every possible combination of tools for the other steps of the pipeline. Therefore, each row shows the grouped average metric for each tool in every step of the pipeline. The network inference methods show the most variation in the global network metrics compared to tools in other steps of the pipeline. [file msystems.00961-22-s0009.pdf]

DC	CC	TA	OP	NI	Average shortest path length	Average clustering	No. of connected components	Modularity	Node connectivity	Degree assortativity coefficient
DB	-	-	-	-	2.066	0.265	27.088	0.387	1.868	0.070
OR	-	-	-	-	1.904	0.273	24.868	-17.060	1.877	0.099
D2	-	-	-	-	2.263	0.267	28.228	0.326	1.789	0.078
DN	-	-	-	-	2.065	0.272	25.132	0.302	1.754	0.093
CR	-	-	-	-	1.937	0.276	24.289	-1.865	2.079	0.097
-	remove bimera	-	-	-	2.070	0.272	25.968	-7.439	1.863	0.086
-	uchime	-	-	-	2.066	0.269	25.874	0.275	1.884	0.089
-	-	NCBI	-	-	2.218	0.262	22.784	-3.683	1.495	0.100
-	-	SILVA	-	-	1.872	0.275	30.716	0.415	2.674	0.074
-	-	GG	-	-	2.086	0.274	24.263	-7.478	1.453	0.088
-	-	-	On	-	1.975	0.252	18.493	-7.194	1.880	0.073
-	-	-	Off	-	2.193	0.291	34.174	0.431	1.867	0.104
-	-	-	-	propr	1.650	0.528	1.000	0.000	10.583	0.036
-	-	-	-	harmonies		0.108	82.633	0.757	0.000	0.340
-	-	-	-	flashweave	3.878	0.079	1.800	0.458	0.383	-0.032
-	-	-	-	cozine		0.090	82.167	0.694	0.000	-0.072
-	-	-	-	spring		0.092	11.767	0.566	0.000	-0.035
-	-	-	-	sparcc	1.747	0.525	1.033	0.009	5.500	-0.011
-	-	-	-	spieceasi		0.076	39.383	0.717	0.000	-0.031
-	-	-	-	pearson		0.457	23.883	0.551	0.000	0.478
-	-	-	-	spearman	1.875	0.551	1.450	0.044	1.117	0.170
-	-	-	-	mldm	2.760	0.130	2.267	-75.648	0.433	-0.028
