# Supplementary material for: Inferring microbial co-occurrence networks from amplicon data: a systematic evaluation
Source: mSystems. 2023 Jun 20;8(4):e00961-22. doi: 10.1128/msystems.00961-22 (PMC10469762; doi:10.1128/msystems.00961-22)
Supplement: Supplemental Text — describing data processing, synthetic interaction data generation, network metrics, P-value merging, additional network variance analysis, and supplemental discussion sections. [file msystems.00961-22-s0010.pdf]

Supplementary Text

Processing the FMT data

Data download and pre-processing

The main biological dataset used in this study was the collection of 16S rRNA sequencing reads from stool samples (healthy and autistic individuals) for a fecal microbiome transplant study [55]. The data containing the 16S sequencing reads (V4 region) was downloaded from Qiita [50] (study ID: 10532). Only runs 2, 3, and 4 were used for the subsequent analysis as these runs consisted of paired-end sequencing data, and run 1 contained single-end data. The sample metadata was updated to contain only BMI, sex, height, weight, and experimental group. This was necessary as two of the network inference algorithms (mLDM and FlashWeave) required information about environmental heterogeneity. However, these environmental correlations were not included in the current analyses.

Processing using the MiCoNE pipeline

The data was then processed using the MiCoNE pipeline starting at the SP step and ending at the NI step with the consensus algorithm. The configuration files (main.nf and nextflow.config) used to run the MiCoNE pipeline as well the details of the pipeline execution (dag, report, timeline and trace) are in the "runs/FMT" directory of the data and scripts repository (https://github.com/segrelab/MiCoNE-pipeline-paper) The results of the pipeline execution for reproducing the analyses in the manuscript are stored on Zenodo.

Processing the mock data

Data download and pre-processing

The mock datasets, mock4, mock12 and mock16 used for this study, were obtained from mockrobiota [56]. Mock 4 is a mock community composed of 21 bacterial strains represented in equal abundances in two replicate samples, and the same strains represented in uneven abundances in two other replicate samples. Mock 12 is composed of 27 bacterial strains containing closely related taxa, the members of which were chosen in part for their well-separated 16S rRNA gene sequences. Some pairs of strains differ by as little as one nucleotide, but all the strains are distinguishable over the sequenced region of the 16S rRNA gene. Mock 16 is a mock community composed of even amounts of purified genomic DNA from 49 bacteria and 10 archaea. The datasets did not require any preprocessing and could be directly used as input to the pipeline

Processing using the MiCoNE pipeline

The data was processed using the MiCoNE pipeline starting at the SP step and ending at the OP step with the filtered taxonomic tables as the final output. The configuration files (main.nf and nextflow.config) used to run the MiCoNE pipeline as well the details of the pipeline execution (dag, report, timeline and trace) are in the ”runs/mock*” directory of the data and scripts repository (https://github.com/segrelab/MiCoNE-pipeline-paper) The results of the pipeline execution for reproducing the analyses in the manuscript are stored on Zenodo.

Interpretation of Unifrac results in the DC step

In Figure S3, we observe that both the weighted and unweighted UniFrac distances are increased for the top 1000 representative sequences, implying that the top representative sequences generated by the different methods are not as similar to each other. Therefore, since the weighted UniFrac distances are lower than the unweighted distances, we conclude that the representative sequences in the middle range of the abundance distribution are those that must be the most similar between the methods.

Open-reference and de novo clustering methods perform the best under the weighted UniFrac metric and the worst (marginally) under the unweighted UniFrac metric (Figure 2C and 2D). This result can be attributed to the large number of low abundance representative sequences that are generated by these methods. Deblur performs poorly under weighted Unifrac and although its performance on the mock4 dataset is the best under unweighted UniFrac, its performance on the other datasets is average. The Deblur method returns a very small number of representative sequences (2388) and this could account for the reason for the high dissimilarity with the other methods as well as irregular performance on the mock data.

Synthetic interaction data

Data generation

The synthetic interaction data for the study were generated using two methods. The first method, “seqtime” [73] utilized generalized Lotka-Volterra (gLV) equations to model the microbial community dynamics and made use of the Klemm–Eguıluz algorithm to generate clique-based interaction networks [26]. We used the seqtime R package to simulate communities with different numbers of species and samples (see Methods for details). The second method, “NorTA” used the Normal to Anything (NorTA) approach coupled with a given interaction network topology to generate the abundance distribution of the microbial community [47]. We used the spieceasi R package to simulate communities with different abundance distributions and network topologies (see Methods for details). The scripts to generate these datasets can be found in the synthetic data and scripts repository (https://github.com/segrelab/MiCoNE-synthetic-data)

Processing using the MiCoNE pipeline

The data was processed using the MiCoNE pipeline using only the NI step with the consensus networks as the final output. The configuration files (main.nf and nextflow.config) used to run the MiCoNE pipeline as well the details of the pipeline execution (dag, report, timeline and trace) are in the ”runs/norta” and ”runs/seqtime” directories of the data and scripts repository (https://github.com/segrelab/MiCoNE-pipeline-paper) The results of the pipeline execution for reproducing the analyses in the manuscript are stored on Zenodo.

Network metrics

In Table S1 we show various global network metrics calculated for each tool in the pipeline. All the networks that make use of a particular tool are grouped together, and the following average metrics are calculated for each group:

1. The average shortest path length describes the average of all the shortest paths in the graph. No number is reported if the graph is not connected, therefore, the results indicate that none of the networks that make use of HARMONIES, COZINE, SPRING, SpiecEasi and Pearson are connected.
2. The average clustering is the average clustering coefficient of the graph. The closer the value is to 1.0, the more densely connected is the graph. We can observe that the networks that use correlation-based methods have the highest values while the direct association based methods have the lowest.
3. The number of connected components is the highest for the direct association based methods and the lowest for the correlation-based methods. In the case of propr, all the networks have only one giant component.
4. The modularity metric is the modularity over all partitions in a graph calculated using a label propagation algorithm [96]. Positive values imply that there are more edges between vertices of the same type than we would expect by chance, and negative implies that there are less. The networks inferred by mLDM report very few edges, and skew the average modularity scores. This could also be an artifact of incomplete convergence of the mLDM algorithm for some combinations.
5. Node connectivity refers to the minimum number of nodes that must be removed from the graph to make it disconnected. We observe that only the networks generated using propr have a high value since most of these networks are connected.
6. Degree assortativity coefficient measures the similarity of connections in the graph with respect to the node degree. Again we observe that the direct association based methods have a negative degree of assortativity, meaning that there are many hubs in these networks. The correlation-based methods have positive values implying that in these networks nodes with similar degrees attach to one another.

A weight threshold of 0.1 and a p-value threshold of 0.05 were applied to each network before the

analysis. All the metrics were calculated using the `networkx` Python package [97].

p-value merging

Fisher [98] proposed that for k independent p-values, each generated by k different methods and denoted by $\bar{P}^i$ (notations are same as used in the "Consensus network and p-value merging" subsection of the Methods). The following will hold true for the statistic Ψ:

$$\Psi = \sum_{i=1}^{k} -2\log\left(\bar{P}^i\right)$$
$$\Psi \sim \chi^2_{2k}$$

Brown [91] extended Fisher's method to dependent p-values by using a re-scaled χ^2 distribution:

$$\Psi \sim c\chi^2_{2f}$$

where, f is the degrees of freedom and c is the scale factor and are given by:

$$f = \frac{\mathrm{E}[\Psi]^2}{\mathrm{Var}[\Psi]} \quad \text{and} \quad c = \frac{\mathrm{Var}[\Psi]}{2\mathrm{E}[\Psi]} = \frac{k}{f}$$

We can calculate $E[\Psi]$ and $Var[\Psi]$ under the null hypothesis that the data are drawn from a multivariate Gaussian with some covariance matrix. We then use these values to parametrize a χ^2 distribution from which the p-value corresponding to $\frac{\psi}{c}$ can be calculated. Furthermore, Brown showed that $\mathrm{E}[\Psi]$ and $\mathrm{Var}[\Psi]$ can be calculated via the following numerical approximation:

$$\mathrm{E}[\Psi] = 2k \quad \text{and} \quad \mathrm{Var}[\Psi] = 4k + 2\sum_{i<j} \mathrm{Cov}\left(-2\log(\bar{P}^i), -2\log(\bar{P}^j)\right)$$

The above formulation was improved by Kost and McDermott [99] by further fitting a third-order polynomial to approximate the covariance

$$\mathrm{Cov}\left(-2\log(\bar{P}^i), -2\log(\bar{P}^j)\right) \approx 3.263\rho_{ij} + 0.710\rho_{ij}^2 + 0.027\rho_{ij}^3 \qquad (8)$$

where, ρ_{ij} is the correlation between method i and method j

Using $E[\Psi]$ and $Var[\Psi]$ we then fit a χ^2 distribution with the parameters c and f. Note that since, in general, f will not be an integer, this should be understood as a Gamma distribution with a shape parameter f, as mentioned by Brown [91]. Using this, we calculate the test ψ and compute the p-value from the CDF of the χ^2 distribution, given in Equation 9. Therefore, the final combined

p-value [92] is then given by:

$$\hat{P}_j = 1.0 - \Phi_{2f}\left(\psi/c\right) \text{ where, } \psi = -2\sum_{i=1}^{k}\log(\bar{P}_j^i) \quad \text{and} \quad \Phi_{2f} = \text{CDF}\left(\chi^2_{2f}\right) \tag{9}$$

The p-value merging and consensus method in MiCoNE (see Methods) uses Equation 8 to estimate the covariance of the p-values and Equation 9 to merge the p-values (obtained from bootstrapping) from the different correlation methods. Note that we do not use Pearson and Spearman methods in the p-value merging step and these algorithms are only used for demonstration and comparison. The combined p-values are used to threshold for significance in the correlation-based networks during the consensus network step.

The JSON network format and network exports

The default format MiCoNE uses for storing the network files is the JSON (JavaScript Object Notation) format which is supported by the Microbial Interaction Network Database (MIND) [54]. The custom JSON schema we have designed is able to store all network-related information pertaining to nodes, links, and the metadata related to the links and datasets. Additionally, MiCoNE also supports exporting of networks into a variety of other formats such as edge lists, GML, and Cytoscape formats. Since we make use of `networkx` [97] for the export functionality, networks can be exported to all formats supported by the package. However, not all the corresponding metadata will be exported appropriately, as most formats do not support this additional metadata. The details of the format and information about importing/exporting it and other network formats can be found in the MiCoNE documentation.

Network variance analysis of stool samples from radiation-exposed bank vole (EMP dataset)

In order to verify the consistency of the network variance analysis, we processed an additional 16S rRNA dataset through the MiCoNE pipeline. Specifically, we used stool samples from radiation-exposed bank vole that were a part of the Earth Microbiome Project (EMP) [74]. We chose a dataset from the EMP because the MiCoNE pipeline inherently supports the EMP amplicon protocol with minimal pre-processing requirements. The data containing the 16S sequencing reads (V4 region) was downloaded from Qiita [50] (study ID: 13114). For the analysis, the paired-end sequencing data extracted from stools samples of radiation-exposed bank vole (named “mousseau”) were chosen from run “EMP500_6-9”. The data was then processed using the MiCoNE pipeline starting at the SP step and ending at the NI step with the consensus algorithm. The configuration files (main.nf and nextflow.config) used to run the MiCoNE pipeline as well the details of the pipeline execution (dag, report, timeline and trace) are in the ”runs/EMP” directory of the data and scripts repository

(https://github.com/segrelab/MiCoNE-pipeline-paper)

Similar to the previous network variance analysis performed in Figure 6, we analyzed the effect of different processing methods on the inferred co-occurrence networks generated using all possible combinations of methods. Figure S8A, shows the percentage of total variation among the co-occurrence networks due to the different steps of the pipeline. The TA step, or more specifically the choice of 16S reference database, contributes the most (57.96%) to the variation in the networks, followed by the OP step (33.48%). These results are very similar to those from the network variance analysis of the FMT dataset (Figure 6A). This implies that the effects of various workflow steps on the inferred networks are fairly consistent across different datasets.

The effects of the different steps of the pipeline on the inferred networks can be visualized through dimensionality reduction. The PCA in Figure S8B shows all possible inferred networks, colored by the tools used in the DC, TA, OP, and NI steps in each subfigure. We observe that the networks largely segregate based on the database used (TA step), and the extent of this separation decreases when the filtering is turned on in the OP step. However, unlike the case of the FMT dataset (Figure 6B), here we observe that the networks that utilize GG and NCBI databases have the variation along similar axes in the 2 dimensional PCA plot. This could imply that the taxonomy assignments returned by these two databases for this dataset, might be more similar to each other compared to those in the FMT dataset. The variation in the networks due to the other workflow steps is consistent with that observed in the FMT data analysis (Figure 6B).

Supplementary discussion

It is worth pointing out some additional, more specific, conclusions stemming from the individual steps of our analysis. The different denoising/clustering methods differ mostly in their identification of sequences that are in low abundance. Hence, they do not have much of an impact on the inferred co-occurrence networks when the sequences of low abundance are removed (Figure 1). Comparison of inferred and expected reference sequences and their abundances in mock community datasets has allowed us to identify DADA2 as the method which best recapitulates the expected sequence composition. For the chimera checking module, we suggest using the remove bimera method since it was developed in conjunction with DADA2 and its performance does not significantly differ from uchime-denovo. For the current work, we have decided to focus on the tools most widely used at the time of the analysis. Some tools which were not as widely used (e.g. dbOTU3 [100]) as well as older popular methods like mothur [101] have not been included in the study but could be added into the pipelines in future updated analyses.

The choice of taxonomy database was found to be the most important factor in the inference of microbial co-occurrence networks, contributing 65.4% of the total variance. The frequent changes in the taxonomy nomenclature coupled with the frequency of updates to the various 16S reference databases create inherent differences [63] in taxonomy hierarchies in these databases. Our analysis revealed that no particular reference database performs better than the others across the different mock dataset benchmarks. The default reference database in the pipeline is currently chosen to be

the GG reference database along with the "Naive Bayes" classifier as the query tool. The reason for our choice stems from the popularity of the GG database [102] in taxonomic studies, which would enable easy comparison across datasets. However, we recommend using the SILVA database for newer studies due to its size and taxonomic comprehensiveness [87] and since GG has not been updated since 2013. Additionally, a particular database might be more appropriate than the rest based on specific requirements. For example, to generate a dataset that is compatible with the MIND platform [54], NCBI, is the most appropriate choice as it guarantees compatibility of taxonomic hierarchy and therefore comparability with other datasets. A detailed study of other taxonomy mapping approaches [103, 104] and integrative approaches combining different databases [105] might offer orthogonal information and better matches. Furthermore, we also enable users to use custom databases [87, 106] with the BLAST and Naive Bayes classifiers that are incorporated into the pipeline (from QIIME2). We suggest that the choice of the database should be made based on possible reported or inferred biases in the representation of given biomes in a specific databases [63, 87], as choosing taxon-specific databases have also been observed to compromise classification [107].

The OP step of the pipeline is second in its contribution to total network variance. This can be attributed to the large number of nodes that are added to the final networks when the filtering is turned off. Additionally, a very large number of nodes also decreases the accuracy of the network inference algorithms for the same sample size [79] and increases the computational complexity [48]. We observe that filtering out taxa that are present in low abundances in all samples increases the proportion of taxa in common between taxonomy tables generated using different reference databases (Figure S4), providing another reason for filtering. We also observe that the reduction in the number of taxa leads to a better agreement in the networks inferred through different methods (Figure 6 and Figure S1). Moreover, filtering is necessary in order to increase the power in tests of significance when the number of taxa is much greater than the number of samples.
